# Correlation of breaking forces, conductances and geometries of molecular junctions

**DOI:** 10.1038/srep09002

**Published:** 2015-03-11

**Authors:** Koji Yoshida, Ilya V. Pobelov, David Zsolt Manrique, Thomas Pope, Gábor Mészáros, Murat Gulcur, Martin R. Bryce, Colin J. Lambert, Thomas Wandlowski

**Affiliations:** 1 Department of Chemistry and Biochemistry, University of Bern, Freiestrasse 3, 3012 Bern, Switzerland; 2 Department of Physics, Lancaster University, Lancaster LA1 4YB, United Kingdom; 3 Research Centre for Natural Sciences, HAS, Magyar tudósok krt. 2, H-1117 Budapest, Hungary; 4 Department of Chemistry, Durham University, South Road, Durham, DH1 3LE, United Kingdom

## Abstract

Electrical and mechanical properties of elongated gold-molecule-gold junctions formed by tolane-type molecules with different anchoring groups (pyridyl, thiol, amine, nitrile and dihydrobenzothiophene) were studied in current-sensing force spectroscopy experiments and density functional simulations. Correlations between forces, conductances and junction geometries demonstrate that aromatic tolanes bind between electrodes as single molecules or as weakly-conductive dimers held by mechanically-weak *π* − *π* stacking. In contrast with the other anchors that form only S-Au or N-Au bonds, the pyridyl ring also forms a highly-conductive cofacial link to the gold surface. Binding of multiple molecules creates junctions with higher conductances and mechanical strengths than the single-molecule ones.

Molecular junctions created by single molecules trapped between two probes enable the creation of nanoscale structures with unique mechanical, electrical, optical and quantum properties^1^,^2^,^3^. A common strategy to form molecular junctions is based on the approach and contact of a sharp nanoprobe to a second probe in the presence of the molecules of interest, followed by the subsequent separation of the probes. This strategy is widely employed in mechanically controllable break junction (MCBJ) or scanning tunnelling microscopy-based break junction (STMBJ)^1^,^2^,^3^ experiments, and in force spectroscopy^4^,^5^,^6^ to characterise electrical and mechanical properties, respectively. In particular, conductance studies with small organic molecules revealed unique correlations between molecular structure and junction conductances. These studies explored the influence of molecular length and conjugation^7^,^8^,^9^,^10^,^11^,^12^, torsion angle^13^,^14^,^15^, electrode material^16^,^17^, and anchoring group^18^,^19^,^20^,^21^,^22^,^23^, on junction conductance. To date, generic force spectroscopy experiments primarily address supramolecular bonds^24^,^25^,^26^,^27^,^28^, and almost no studies of mechanical properties of covalently-bound junctions were reported^4^.

Few research groups employed current-sensing atomic force microscopy (CSAFM) technique to simultaneously characterise the conductance of single-molecule junctions and the forces acting on them^29^,^30^,^31^,^32^,^33^,^34^,^35^,^36^. The force acting on a molecular junction during its elongation represents various interactions, and varies even in the absence of structural rearrangements. The specificity of a junction is rather reflected by a distribution of forces required to break it^4^,^5^,^6^. Various methods were applied in biophysical research to distinguish specific breaking events from so-called “non-specific” ones^26^,^27^,^28^. In the CSAFM approach, it is possible to take advantage of the simultaneously recorded conductance to detect the formation and breaking of molecular junctions. Until now this approach has not been applied to address electromechanical properties of non-covalently bound intermolecular junctions, such as those based on *π* − *π* stacking^20^,^22^,^37^,^38^, hydrogen bonding^39^ or host-guest interactions^40^.

In this work, we carried out CSAFM experiments^33^,^41^,^42^ in solution and evaluated conductances and breaking forces at the different stages of elongation of Au-molecule(s)-Au junctions formed by symmetric tolane-type molecules (Figure 1) with two pyridyl (1,2-di(pyridin-4-yl)ethyne, PY2), dihydrobenzothiophene (1,2-bis(2,3-dihydrobenzo[*b*]thiophen-5-yl)ethyne, BT2), amine (4,4′-(ethyne-1,2-diyl)dianiline, NH_2_2), nitrile (4,4′-(ethyne-1,2-diyl)dibenzonitrile, CN2), and thiol (4,4′-(ethyne-1,2-diyl)dibenzenethiol, SH2) anchoring groups. Their conductance was recently characterised in STMBJ and MCBJ experiments^20^,^22^. Unlike in other reports, where a single value of the breaking force was determined for one type of molecular junction^29^,^30^,^32^,^35^,^36^, we have successfully tracked junction breaking forces at different stages of the elongation process and correlate them with the conductance and geometry of the molecular junction. We also investigated molecular junctions formed by an asymmetric compound with only one thiol anchoring group (4-(phenylethynyl)benzenethiol, SH1) to explore the electromechanical characteristics of *π* − *π* stacked junctions^37^,^38^. To obtain insight into the experimental results, complementary density functional theory (DFT) based simulations of the junction breaking process were carried out.

## Results

### Current-sensing AFM experiments

We monitored the creation of probe-sample contacts and the stochastic formation and breaking of molecular junctions upon their elongation by measuring the current *I* flowing between the two electrodes upon the application of constant potential difference *E_bias_* and the force *F* acting on the cantilever as a function of the elongation distance *z*. As the junction conductance *G* = *I*/*E_bias_* varies over many orders of magnitude during the elongation, we present it in the normalised logarithmic scale log(*G*/*G*_0_), where *G*_0_ = 77.5 *μ*S is the quantum of conductance. *G*_0_ also corresponds to the conductance of a gold junction with a single atom in the cross-section. Examples of individual elongation traces for PY2 junctions are described in Supplementary Note 1. Among all studied tolanes, BT2 and PY2 form molecular junctions with the high probability; CN2, NH_2_2 and SH1 with the low one and SH2 represents an intermediate case (Supplementary Note 2).

**Figure 2:**
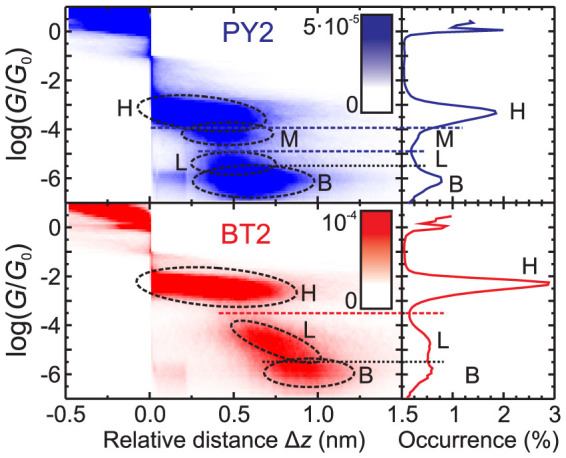
2D histograms of log(*G*/*G*_0_) versus Δ*z* (left) and 1D histograms of log(*G*/*G*_0_) (right) constructed from all data points of the measured elongation traces for the molecular junctions formed by BT2 and PY2. The occurrence of data points in the 2D (size 0.01 nm × 0.1) and 1D (size 0.1) bins is given by the colour scale and by the solid lines, respectively. The dashed ellipses in the 2D histograms and letters in 1D histograms mark features of molecular junctions with high (H), medium (M) and low (L) conductance as well as features of the baseline conductance (B). The latter are separated from the molecule-specific features by a dotted horizontal line. The dashed horizontal lines correspond to the positions of borders between the conductance ranges log(*G_HM_*/*G*_0_), log(*G_ML_*/*G*_0_) for PY2, as well as log(*G_HL_*/*G*_0_) for BT2 (Supplementary Table 2).

Figure 2 shows 1D and 2D histograms of log(*G*/*G*_0_) constructed from all measured traces for the highly stable BT2 and PY2 junctions. All traces were aligned in a common distance scale by setting the first point with the conductance *G* < 0.1 *G*_0_ to the Δ*z* = 0. The corresponding plots for NH_2_2, CN2, SH2 and SH1 are presented in Supplementary Fig. 3. All histograms display a region of high data density at Δ*z* < 0 and log(*G*/*G*_0_) > 0 (2D), as well as sharp peaks at log(*G*/*G*_0_) ≈ 0 (1D). These features are characteristic of the formation and breaking of (single-atom) Au-Au contacts^33^. Horizontally aligned clouds (2D) in the range log(*G*/*G*_0_) < −5.5 and broad peaks with a maximum at log(*G*/*G*_0_) ≈ −6 (1D) correspond to the “baseline” conductance (B) measured in the absence of an electrical contact between the two gold electrodes, i.e. when the junction is completely broken. Conductance values in the interval −5.5 < log(*G*/*G*_0_) < −0.5 reflect the properties of specific molecular junctions.

Junctions formed by PY2 reveal one sharp peak of high molecular conductance H (−3.9 < log(*G*/*G*_0_) < −2.0) and two less pronounced shoulders representing the medium (M) and low (L) conductance ranges in the 1D histogram^20^,^22^. More details can be extracted from the 2D histogram. A medium conductance feature appears below the H cloud in the range −4.7 < log(*G*/*G*_0_) < −3.9. A low conductance feature is found at −6.0 < log(*G*/*G*_0_) < −5.0. The latter partially overlaps with the baseline conductance.

The histograms for BT2 display two well-separated high (H, −3.3 < log(*G*/*G*_0_) < −1.5) and low (L, −5.5 < log(*G*/*G*_0_) < −4.0) conductance ranges. The molecular junctions of NH_2_2 and SH2 also reveal two conductance ranges H and L, while CN2 and SH1 display only one conductance feature (Supplementary Fig. 3). The conductances in each range appears to decrease steadily with the stretching length Δ*z*. A most probable junction conductance can be estimated from the 1D histograms in every case (Table 1). The conductance results obtained in the present CSAFM experiments are in a good agreement with those reported in our previous STMBJ and MCBJ studies^20^,^22^ (Supplementary Table 3).

### Experimental breaking forces

**Figure 3:**
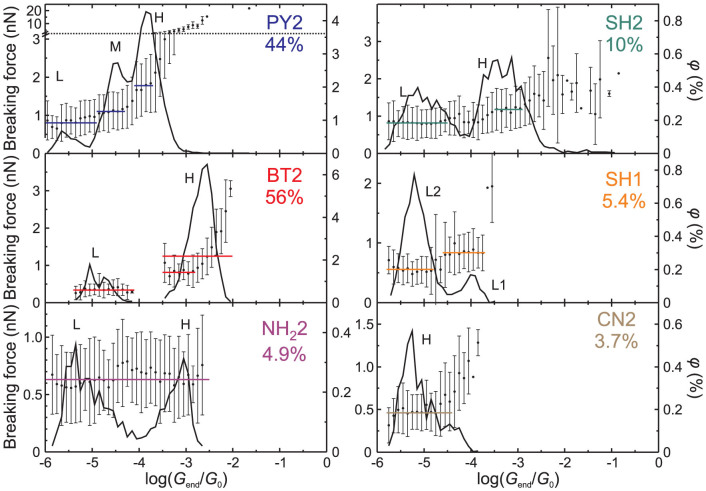
Experimental breaking force *F_b_*_,*e*_ (circles, scale on the left) and its standard deviation (error bars) for molecular junctions formed by tolanes with different anchoring groups as a function of the logarithm of normalised junction conductances prior to the breaking event log(*G_end_*/*G*_0_). The solid curves (scale on the right) represent the percentage of traces *ϕ* used to determine *F_b_*_,*e*_ at each value of *G_end_*. The peaks of *ϕ* are attributed to the breaking of junctions with conductance in the indicated range. The sum values of *ϕ* are shown in the panel labels. The horizontal lines represent the mean values of the breaking forces *F_b_*_,*e*_ in the corresponding ranges of *G_end_* (Table 1).

The mechanical properties of molecular junctions were characterised by determining their breaking forces *F_b_* as a function of their conductance prior to the breaking *G_end_* (Supplementary Note 3). We found that due to the stochastic variation of the junction's structure, different stages of the elongation process are better identified by *G_end_* than by the extension length Δ*z*. Figure 3 displays the experimental mean breaking force *F_b_*_,*e*_ as well as the percentage of analysed traces *ϕ* vs. log(*G_end_*/*G*_0_) for all studied tolanes. *ϕ* equals the number of traces with given *G_end_* divided by the total number of measured traces. Due to the different junction formation probability and a restriction to low-noise junctions, the highest percentage of traces was analysed for BT2 and PY2, the lowest for SH1, NH_2_2 and CN2, and an intermediate one for SH2.

As a general trend, as *G_end_* decreases, *F_b_*_,*e*_ displays alternating intervals of decreasing and rather constant values, with the latter coinciding with the positions of peaks in *ϕ*. The dependence of the percentage of analysed traces *ϕ* on log(*G_end_*/*G*_0_) closely resembles the shape of the 1D histogram of log(*G*/*G*_0_). We assign the peaks of *ϕ* to the conductance of stable configurations of junctions within the indicated ranges. Since *G_end_* is lower than junction conductances encountered during an elongation process, the peaks of *ϕ* are located at slightly lower values than the positions of the most probable conductance peaks in the respective 1D histograms. For the ranges of conductances log(*G_end_*/*G*_0_) corresponding to rather constant *F_b_*_,*e*_, as indicated by the horizontal lines in Fig. 3, the vertical positions of the latter indicate the average experimental breaking forces *F_b_*_,*e*_ (Table 1).

For PY2, the three mean breaking forces, 1.8 ± 0.7 nN, 1.1 ± 0.5 nN and 0.8 ± 0.4 nN, were estimated from values in the regions near the H, M and L peaks of *ϕ*, respectively (Fig. 3). The results of the PY2 high-conductance junctions are in agreement with breaking forces ranging between 1.5 and 1.9 nN, as reported by Aradhya et al.^34^ for a series of molecules with pyridyl anchoring groups. These values are higher than the breaking force of a single Au-Au contact at the same force loading rates, *F_b_* ≈ 1.5 nN^33^. Several groups reported a breaking force of *F_b_* ≈ 0.8 nN for Au-dipyridyls-Au junctions and attributed this value to the breaking of a single-molecule junction^29^,^30^,^34^.

For BT2, Fig. 3 reveals two well-separated peaks of *ϕ*, with mean breaking forces *F_b_*_,*e*_ of 1.2 ± 0.6 nN (H) and 0.3 ± 0.2 nN (L). The breaking force in the H range decreasing at low values of *G_end_* to 0.8 ± 0.3 nN.

For SH2, the mean values of *F_b_*_,*e*_ show a rather continuous transition between the L and the H peaks in *ϕ*, with breaking forces of 1.2 ± 0.5 nN and 0.8 ± 0.4 nN, respectively. The first value is in a good agreement with data of Frei et al.^35^, who obtained for Au-1,4-butanedithiol-Au junctions breaking forces ranging between 1 and 1.2 nN. The latter and the present experimental studies are distinctly different from most other reports for thiol-based molecular junctions. It is often believed that the S(H)-Au bond breaks by pulling out Au atoms from the electrodes^4^,^29^,^31^,^36^ with *F_b_* reaching values of 1.5 nN typical for clean atomic contacts. Complementary experiments with SH1 revealed two regions L1 (minor) and L2 (major) with mean breaking forces decreasing from 0.8 ± 0.3 nN to 0.5 ± 0.3 nN.

NH_2_2 junctions exhibited a single force *F_b_*_,*e*_ = 0.6 ± 0.3 nN in the range −5.9 < log(*G_end_*/*G*_0_) < −2.5, which covers both H and L conductance ranges. This result is in good agreement with breaking forces of 0.5 to 0.7 nN, which were reported previously for molecular junctions formed by aliphatic and aromatic diamines^30^,^35^. In the case of CN2 only a small number of molecular junctions could be created, which led to a single mean breaking force converging to 0.5 ± 0.2 nN at low *G_end_*.

### Simulated elongation sequences

The experimental CSAFM studies were complemented with numerical simulations of the elongation process for the corresponding molecular junctions composed of either one or two molecules trapped between gold leads. The latter case allowed us to explore theoretically the formation of configurations with multiple molecules bound to both electrodes as well as junctions with *π* − *π* stacked molecules.

**Figure 4:**
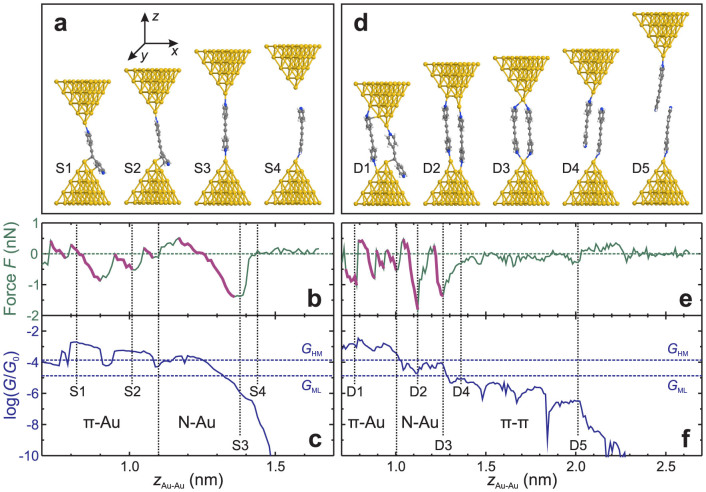
Representative structures (a, d), coordinate axes (a), theoretical loading force *F* (b, e) and logarithm of the normalised conductance log(*G*/*G*_0_) (c, f) of single (a–c) and double (d–f) molecule junctions of PY2 as a function of the electrode separation *z_Au_*_–*Au*_. The vertical dotted lines mark positions corresponding to the structures S1–S4 and D1–D5, and transitions between configurations with different type of coupling. The thick parts of the force traces illustrate the elastic stages of junction elongation. The horizontal dashed lines in the conductance panels indicate the positions of experimental borders between the high/medium (*G_HM_*) and the medium/low (*G_ML_*) conductance ranges.

For PY2, Figs 4a–4c show the theoretical loading force *F* and logarithm of the normalised conductance log(*G*/*G*_0_) of a single-molecule (S) junction as a function of the electrode separation *z_Au_*_–*Au*_ (the distance between the centres of the apex atoms of opposing gold pyramids). The computed absolute distance scale *z_Au_*_–*Au*_ is approximately related to the experimental relative distance scale Δ*z* by *z_Au_*_–*Au*_ = Δ*z* + 0.5 nm, where 0.5 nm is a typical snap-back distance of the electrodes after breaking of the Au-Au contact^22^. Figure 4a illustrates four characteristic configurations of the PY2-S junctions labeled S1 to S4. The computed loading force *F* and conductance traces are shown in Fig. 4b and 4c. At low *z_Au_*_–*Au*_, the molecule is attached by the *π*-systems of the pyridyl rings to the surface of at least one gold pyramid (*π*-Au coupling), as illustrated by structures S1 and S2. As the junction evolves, a molecule in a stable configuration experiences an elastic elongation with |*F*| increasing linearly with electrode separation, as shown by parts of force trace marked by a thicker line in Fig. 4b. At small *z_Au_*_–*Au*_, this sliding produces saw-tooth-like features in *F*. Upon reaching a certain force threshold (position S2), the junction experiences an inelastic deformation and adopts a new stable geometry. Structurally, the *π*-system(s) of PY2 slide across the gold pyramids without losing contact. As *z_Au_*_–*Au*_ approaches the molecular length, the molecule jumps into the gap and binds to the apex atoms of both pyramids forming coordination bonds between the nitrogen atoms of the pyridyl groups and the gold atoms (N-Au coupling). Further pulling of this configuration leads to a relatively long elastic stage until one Au-N bond dissociates (structure S4). Breaking of the junction results in a rapid decrease of the conductance and the relaxation of the loading force towards zero. The observed residual conductance is attributed to through-vacuum tunnelling.

We also simulated the junction formation and breaking process of configurations with a PY2 dimer (D) attached to gold leads (Figs 4d–4f). In a PY2-D junction, the two PY2 molecules assume a range of configurations similar to that of a PY2-S junction, as shown in Fig. 4d. Structure D1 represents a typical configuration with *π*-Au coupling, which transforms upon elongation to a configuration with both molecules being coupled to different atoms of the gold electrodes via N-Au bonds (structure D2). The sliding of anchoring groups across the gold surfaces during the elongation again leads to saw-tooth-like features in *F* (Fig. 4e). Upon increase of *z_Au_*_–*Au*_ both molecules bind to the same apex atoms (structure D3). Further elongation leads to the breaking of one Au-N bond for each molecule and to the formation of a non-covalent “dimer junction” (structure D4), in which two molecules are held together by *π* − *π* stacking. This transition is manifested as a distinct force relaxation and as a decrease of junction conductance by more than one order of magnitude. Further junction elongation leads to a sliding of the molecules across each other, accompanied by a relatively small fluctuation of the force and a steady decrease of the conductance until the complete elongation of the dimer junction. The subsequent breaking of the dimer junction (D5) leads to a small force relaxation and to a rapid drop in conductance.

Simulated elongation processes for BT2, NH_2_2, CN2 and SH2 show similar results (Supplementary Figs. 7–9). The *π*-electron-rich tolanes are predicted to form stable dimers, irrespective of the nature of the anchoring group. This conclusion is in agreement with our recent DFT calculations^20^,^22^.

### Assignment of the experimental conductance ranges

In agreement with our previous results^20^,^22^, we assign the H range for BT2, SH2, NH_2_2 as well as the single conductance range of CN2 to covalent junctions, i.e. those with at least one molecule bound to both electrodes. PY2 demonstrates three experimental conductance ranges and thus represents a special case. Our simulations (Figures 4c and 4f) indicate that PY2 forms covalent junctions with two coupling arrangements to the electrodes (*π*-Au and N-Au), which exhibit distinctly different conductance. We associate them with experimental high and medium conductance (Fig. 2). BT2, NH_2_2, SH2 and CN2 also form structures with an aromatic ring attached to gold pyramids (see Supplementary Figs. 7–9 and Supplementary Movies 1–6). However, only for PY2 the junction conductance is significantly different in *π*-Au and N-Au coupling arrangements. We attribute this difference to steric effects of the anchoring groups, which prevent a sufficiently close approach of the phenyl ring to the gold surface in tolanes other than PY2.

To verify this hypothesis, we analysed geometry of single-molecule junctions during the simulated elongations (Supplementary Note 4). Supplementary Table 5 shows that during the sliding phases such as S1 and S2 of Fig. 4a, the average distance of the pyridyl ring from the electrode surface is significantly lower than that of the phenyl rings in other tolanes. Also the distance between the N atom in PY2 and the closest gold atom during the sliding stage is significantly higher than the value for a relaxed bond (Supplementary Table 4), and the N-Au distance decreases to the latter after transition to the N-Au coupling. For other tolanes the aromatic ring is far from the gold atoms during the sliding stage, and the electronic coupling with gold occur primarily via the anchoring group^22^. Therefore for the latter the transition to single atom coupling does not lead to a significant modification of the electronic structure of the junction, and, as a consequence, its conductance. Both types of geometry thus contribute to the experimental H range.

The comparison of theoretical conductance of *π*-*π*-stacked dimers (Fig. 4, Supplementary Figs. 7, 8) with the experimental data (Fig. 2, Supplementary Fig. 3, Supplementary Table 2) allows us to assign experimental low-conductance ranges of PY2, BT2, SH2 and NH_2_2 to dimer junctions formed by two molecules, each bound to one electrode only and held together by *π* − *π* stacking interactions (c.f. structures D4 and D5 in Fig. 4). The existence of this type of junction was experimentally demonstrated by MCBJ and STMBJ experiments with aromatic molecules bearing only one anchoring group^37^,^38^. Also our experiments demonstrate that the compound SH1 bearing only one thiol group forms molecular junctions with conductance similar to that of the low-conductance range of SH2 (Supplementary Fig. 3). Further support for this assignment arises from the concentration-dependent conductance studies. STMBJ experiments carried out in solutions with BT2 of variable concentrations ranging from 0.1 *μ*M to 1 mM demonstrated identical H conductance features in the 1D and 2D histograms, but a gradual reduction of the number of L conductance junctions upon the decrease of the concentration, until they disappear completely at the lowest studied concentration^22^. This trend provides strong experimental evidence that the formation of low-conductance junctions requires the presence of more than one molecule.

The qualitative difference between covalent and dimer junctions is well illustrated by the 2D conductance histogram of BT2 (Figure 2): the conductance of dimer junctions displays a much stronger dependence on the relative distance Δ*z* than the conductance of covalent junctions. This difference is accounted for by the evolution of the junctions' geometry during their elongation (see above). The elongation of covalent single-molecule junctions proceeds via the step-wise sliding of the anchoring group across the gold surface, which did not change the junction's conductance significantly. On the other hand, the elongation of a dimer junction results in a sliding of the molecules across each other, which decreases the overlap between the *π*-systems of two molecules with the increase of Δ*z* and leads to the decrease of the junction's conductance^37^.

### Simulated breaking forces

Calculated elongation traces such as those in Fig. 4 allowed us to estimate theoretical terminal breaking forces *F_t_*_,*t*_, which we define to be the maximum loading force attained just before the junction breaks (Supplementary Tables 7, 8). This is the approach applied in the previous studies^30^,^34^,^35^. In contrast to such studies, we employed a strategy which allows computation of breaking forces *F_b_*_,*t*_ for every simulated junction geometry. Under experimental conditions, thermal motion potentially enhanced by the local heating due to the passing current, mechanical noise, irregular electrode shapes and geometrical barriers created by solvent molecules could force the molecule to detach before reaching the maximum possible junction extension. Furthermore, the transitions from one local minimum of energy to another may also release kinetic energy and promote the junction breaking.

To determine theoretical breaking forces of single-molecule junctions at each stage of the junction elongation, we induced breaking by displacing the lower gold pyramid along the *x*-axis (i.e. perpendicular to the *z*-axis joining the apex gold atoms of opposing electrodes, Fig. 4a) by 0.01 nm in a sequence of 30 steps. Without further geometry relaxation, we calculated the magnitude of the total Hellmann–Feynman force acting on the lower pyramid for each displacement. The *z*-component of the force vector with maximum magnitude was then taken as the breaking force *F_b_*_,*t*_ of the junction. In the case of double-molecule junctions we displaced the lower gold pyramid and the right molecule. The breaking force was then calculated in the same way as above from the force vector on the lower gold pyramid and the right molecule.

**Figure 5:**
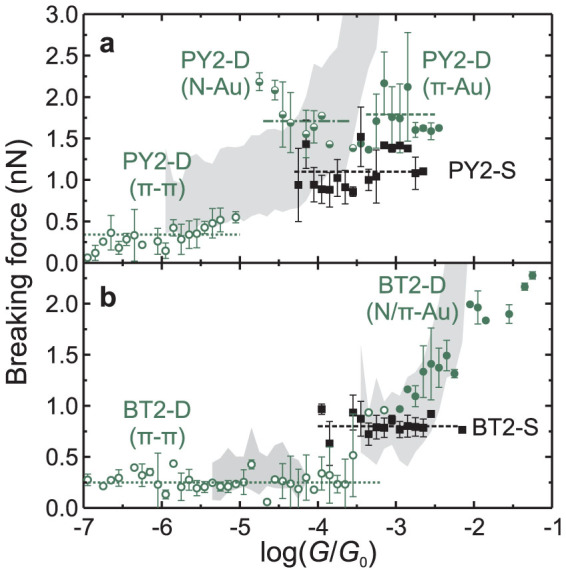
Mean breaking force *F_b_*_,*t*_ (symbols) and its standard deviation *F_b_*_,*t*,*err*_ (error bar) for simulated junctions of PY2 (a) and BT2 (b) as a function of the logarithm of the normalised conductance log(*G*/*G*_0_). Squares and dashed lines represent *F_b_*_,*t*_ and their mean values for single-molecule junctions. The open circles and dotted lines correspond to *F_b_*_,*t*_ of *π* − *π* stacked dimer junctions and their mean values. PY2: The full circles and dashed line correspond to double-molecule junctions with *π*-Au coupling and their mean value. The half-full circles and dot-dashed lines correspond to the double-molecule junctions with N-Au coupling and their mean values. BT2: The full circles correspond to double-molecule junctions with at least one molecule bridging both electrodes. The grey bands mark the experimental *F_b_*_,*e*_ ± *F_b_*_,*e*,*err*_ vs. log(*G_end_*/*G*_0_) dependences, as shown in Fig. 3.

The resulting values of the breaking force *F_b_*_,*t*_ were grouped into 0.1-wide bins of log(*G*/*G*_0_) and the mean value as well as the standard deviation of *F_b_*_,*t*_ were calculated for each group. For PY2 and BT2, the squares and the circles of Fig. 5 correspond to theoretical results for *F_b_*_,*t*_ vs. log(*G*/*G*_0_) for S and D junctions. For comparison with experiment, the shaded regions of Fig. 5 show the distribution of corresponding experimental results in Fig. 3. Results for NH_2_2 are presented in Supplementary Fig. 11. Table 1 lists the mean breaking forces for the different types of simulated junctions that were attributed to the experimental conductance ranges.

### Assignment of the breaking forces

Despite the different conductance of the *π*-Au and N-Au coupling arrangements of single-molecule junctions of PY2, Fig. 5a shows no clear difference between breaking forces calculated for them (≈1.1 nN, Supplementary Table 6). The solid and half-solid circles of Fig. 5a correspond to PY2 dimer junctions (PY2-D) with *π*-Au and N-Au coupling; mean breaking forces calculated for them are 1.8 nN and 1.7 nN, respectively.

We propose the following interpretation of experimental *F_b_*_,*e*_ vs. log(*G_end_*/*G*_0_) dependence for PY2. Upon electrode separation the junctions with conductance in experimental H range and the most probable breaking force of 1.8 nN are formed first. We attribute them to the simulated double-molecule junctions with *π*-Au coupling, such as the structure D1 in Fig. 4, whose breaking force is 1.8 nN (Table 1). At higher electrode separations we found junctions with conductance in experimental M range and a breaking force of 1.1 nN. They are attributed to single-molecule junctions with N-Au couplings to the electrodes. Their formation under these conditions appears to be more probable than the simultaneous binding of few molecules to both electrodes. When the electrode separation exceeds the length of the PY2 molecule, the *π* − *π* stacked dimer junctions are formed by two PY2 molecules bound to opposite electrodes. Both their conductance and breaking force are significantly lower than those of covalent junctions.

We note that in Ref. 34 the different breaking forces of junctions formed by molecules with pyridyl anchoring groups were associated with different coupling of a single molecule in a junction. By contrast, our results demonstrate that the breaking force of PY2-S junctions (1.1 nN) does not depend on the coupling type (however the latter affects the junction conductance). A simultaneous binding of two molecules is required to obtain a higher breaking force of 1.8 nN. Moreover, we note that the simulation with one or two PY2 molecules did not produce junctions with very high conductances and very high breaking forces, corresponding to the *G_end_* range above the H peak in Fig. 3. Considering their low experimental formation probability, we attribute data in this range to the contacts formed by more than two molecules connecting electrodes in one or in few junctions created between the AFM probe and the sample.

The theoretical breaking forces of single-molecule junctions of BT2 (BT2-S, squares in Fig. 5b) is independent of the coupling type and averages to 0.8 nN. BT2 junctions with two molecules bound at both ends to opposing electrodes (BT2-D, solid circles in Fig. 5b) show a single sequence of breaking force decreasing with the decrease of junction conductance. The theoretical conductance of both BT2-S and BT2-D junctions corresponds to the experimental H range. The corresponding breaking forces build single *F_b_*_,*t*_ vs. log(*G*/*G*_0_) dependence, almost quantitatively reproducing the experimental data. Therefore, we assign the sections of *F_b_*_,*e*_ vs. log(*G_end_*/*G*_0_) dependences, characterised by decreasing values of *F_b_*_,*e*_ and their leveling off with the decrease of *G_end_* (Fig. 3), to contributions of junctions with two and one BT2 molecule bound between the gold electrodes. The formation of covalent junctions with more than two molecules appears to be inhibited for BT2 due to steric effects of the anchoring group. Similar to PY2, the *π* − *π* stacked dimer junctions exhibit lower conductance and lower breaking force than the covalent junctions.

Based on an analogy with BT2 and PY2, we assign the breaking force *F_b_*_,*e*_ = 1.2 nN obtained for SH2 in the H conductance range to the breaking of covalent junctions, and the low-probability junctions with higher conductance and higher breaking force to the contribution of junctions with multiple molecules bound between two electrodes. For CN2, the experimental *F_b_*_,*e*_ vs. log(*G_end_*/*G*_0_) dependence (Fig. 3) is similar to that of BT2 in the high conductance range, suggesting that CN2 might also form single-molecule and double-molecule junctions, the latter being stronger and more conductive.

Overall the theoretical breaking forces for BT2-S and BT2-D (Fig. 5b) almost quantitatively reproduce the experimental data and the results for PY2 (Fig. 5a) are in a good qualitative agreement. Experimentally, these are the tolanes forming junctions with the highest probabilities. Qualitatively, we found the same trend of experimental breaking forces in H conductance range and of theoretical terminal breaking forces of single-molecule junctions (*F*(SH2) > *F*(PY2) > *F*(BT2) > *F*(NH_2_2), with the exception of CN2), provided one assumes deprotonation of thiol groups of SH2 upon binding to the gold leads (Supplementary Table 7).

Experimental breaking forces of *π* − *π* stacked dimer junctions follow the order *F*(PY2) ≈ *F*(SH2) > *F*(SH1) ≈ *F*(NH_2_2) > *F*(BT2) (Table 1). The highest and lowest values obtained for PY2 and BT2 dimers correlate well with the theoretical binding energies and with the theoretical breaking forces (Supplementary Table 8). Structurally, we attribute the above difference between the tolanes, with PY2 and BT2 being extreme cases, to steric effects of the anchoring groups. As the N atoms in PY2 constitute parts of the aromatic rings, unhindered *π*-stacking of the two PY2 molecules is allowed, whereas *π*-stacking of BT2 is considerably hindered by the large dihydrothienyl rings, which are not parts of the *π*-electron systems.

Finally, we found that the theoretical breaking forces for *π* − *π* stacked double-molecule junctions of NH_2_2 are distinctly lower than those of covalently-bound single or double-molecule junctions (Supplementary Fig. 11). In contrast, the experimental breaking forces of NH_2_2 (Fig. 3) show no dependence on the conductance. Empirically, we suggest that both single-molecule and dimer junctions break via the rupture of the same bond, namely the NH_2_-Au bond, rather independently of the junction geometry. This hypothesis is also supported by the value of *F_b_*_,*e*_ = 0.6 nN obtained for NH_2_2 in both conductance ranges, which is lower than *F_b_*_,*e*_ for single-molecule and for *π*-stacked junctions of PY2 and SH2. The employed DFT method, which is actually not particularly suitable for the estimation of intermolecular interactions, seems to estimate rather well breaking forces for covalently-bound junctions, but underestimate breaking forces for *π*-stacked dimers. This might be the reason why we did not observe in simulations the detachment of the NH_2_-Au bond upon breaking of a junction with two *π*-stacked NH_2_2 molecules.

## Discussion

In our combined experimental and theoretical study of electrical and mechanical properties of metal-molecule-metal junctions we develop the picture of the interplay between structure, conductance and mechanical properties in nanoscale systems to a level of detail which would not be accessible by studying conductances or forces alone. Experimentally, we demonstrated how the junction conductance, accessible in CSAFM, can be utilised to identify formation of molecular junctions and to track their breaking force during the junction elongation. The latter is a key process in experimental approaches aiming at probing mechanical (force-distance spectroscopy) or electrical (break junction-like techniques) properties of nanojunctions. Theoretically, we considered for the first time the binding of more than one molecule in a junction. The computation of theoretical breaking forces at every elongation step is the first of its kind; only breaking at the maximum junction length was analysed theoretically before.

The investigated compounds have a basic aromatic *π*-system of 1,2-diphenylethyne (tolane) and various anchoring groups (thiol, pyridyl, amine, nitrile and dihydrobenzothiophene). They produce junctions of two or three clearly distinguishable structural types. At low electrode separations one or many molecules may bind to both electrodes, forming covalent junctions. The more molecules connect electrodes, the higher is the conductance and the breaking force of the junction. Uniquely, the pyridyl anchoring group can also bind to gold via a highly-conductive cofacial link between the *π*-system of the aromatic ring and vacant orbitals of the gold electrode. With the increase of the electrode separation, the molecules in the junction adapt new geometries and/or detach, ultimately until a single-molecule junctions is formed. These processes cause the decrease of the conductance and of the breaking force. When the electrode separation exceeds the length of a single molecule, *π*-electron-rich molecules create non-covalent junctions. They are formed by two molecules, each attached to one electrode only, which are held together by the interaction between the *π*-electrons of their aromatic systems. The non-covalent junctions exhibit lower conductance and breaking force than the covalent ones.

## Methods

**Synthesis** of the bifunctionalised tolanes PY2, BT2, NH_2_2, CN2 and SH2 was carried out as described earlier^20^,^22^,^43^. Compound SH1 was synthesised and purified as the thioacetate derivative as reported in Ref. 43.

**Current-sensing force spectroscopy experiments** were carried out using a commercial SPM setup (PicoPlus 5500, Agilent Technology) and a lab-build circuitry as described in Ref. 33. Molecular junctions were formed by contacting a gold sample with a gold-coated AFM probe (PPP-NCSTAu, Nanosensors) in decane containing 0.2 mM of the respective compound and subsequently pulling the latter away from the surface with a rate of *r* = 10 nm·s^−1^. Junction conductance was calculated from the current flowing between the two electrodes at constant applied bias voltage 0.13 V. The spring constant of the cantilevers employed was *k* = 12.5 ± 1.7 N·m^−1^, which leads to a constant force loading rate *r_F_* ≈ 125 nN·s^−1^. The numbers of measured traces were: 16242 (PY2), 4690 (BT2), 29717 (NH_2_2), 33415 (CN2), 23417 (SH2), 16082 (SH1).

**DFT calculations** were performed using the GGA exchange correlation potential with PBE parametrisation, 300 Ry mesh cutoff, double-*ζ*-polarised basis set, force tolerance of 0.01 eV/Å. Geometry optimisation was carried out using the DFT package SIESTA^44^. In case of the isolated molecules a sufficiently large unit cell was employed.

The elongation process was simulated by computing optimised junction geometries and corresponding conductances and forces of a series of structures with increasing electrode separation. The junction was modelled with an *extended molecule* composed of one or two molecules attached to two (111) directed pyramids of 35 gold atoms oriented as mirror reflections on a common main axis *z* (*D*_3*d*_ symmetry) as discussed in Ref. 22. Initially the molecules were placed between the pyramids so that their aromatic rings were parallel to the gold surface ≈ 0.2 nm from the surface. The shortest electrode separation was typically 0.6 nm less than the molecular lengths. Subsequent structures with larger separations were constructed by shifting the upper gold pyramid along the *z*-axis in steps of 0.01 nm. In total 100 or 200 structures were employed for single and double-molecule junctions, respectively. To avoid symmetry artefacts during geometry optimisation, the molecules were shifted by 0.1 nm from the middle point towards one of the pyramids, which we refer to as the lower electrode.

The initial geometry construction was followed by geometry relaxation, in which the first two layers at the base of each pyramid were fixed, and the remaining three layers of each pyramid (closest to the molecule) and the molecule were allowed to relax. After geometry optimisation the electrode separation changed slightly from the initial value to a final value that we refer to as *z_Au_*_–*Au*_.

Conductance values for all optimised geometries were obtained by DFT and quantum transport calculations using the GOLLUM extension of the SMEAGOL package^45^,^46^ as described in our recent works^20^,^22^. The Hellmann–Feynman forces on each atom in a junction were computed for the optimised geometry, and the *z*-component of the total force vector acting on the upper gold pyramid was taken as the loading force *F* acting on a junction at a given electrode separation.

Due to the unclear mechanism of thiol adsorption on gold, we considered in the case of SH2 bonding without and with the formation of a chemical bond (physisorption and chemisorption). We performed two types of simulations: with the thiol anchoring groups -SH (results labeled as SH2) and with the thiyl anchoring groups -S (results labeled as S2), i.e. the terminal hydrogen atom was removed in the latter case. The terminal breaking force of the single-molecule junction of S2 corresponds better to experimental results, but the computation of breaking forces according to the described procedure was not undertaken. The major challenge is to account for the formation of a free thiyl radical upon breaking of the S-Au bond. Under experimental conditions we do not expect a free radical to exist in condensed media. Instead, it may pull out a gold atom from the electrode or become passivated by some environment-specific mechanism.

In the case of CN2, we did not perform a new elongation simulation, but calculated loading forces for the geometries of the single-molecule junction obtained in simulations presented in Ref. 22.

Supplementary Movies 1–6 shows the geometry, the conductance, the loading force and the breaking force for single and double-molecule junctions of BT2, PY2 and NH_2_2 for all electrode separations *z_Au_*_–*Au*_. In the video files, the upper panel shows the junction geometry. The bottom-left panels show the logarithm of the normalised junction conductance (blue circles) and the pulling force (green curves). The red lines and the cyan paraboloid curves are fitted guides to the eye to demonstrate the correlation between the simulated conductance and force traces. The moving vertical black line marks the electrode separation *z_Au_*_–*Au*_ for the current junction geometry. In the bottom-right panel the calculated breaking force is plotted as a function of junction conductance. The vertical and horizontal lines mark conductance and breaking force for the current geometry.

## Author Contributions

K.Y. carried out CSAFM experiments. I.V.P. analysed experimental data and prepared the manuscript using feedback from other authors. D.Z.M. and T.P. performed DFT calculations under the supervision of C.J.L. G.M. built employed instrumentation. M.G. synthesised investigated compounds under the supervision of M.R.B. T.W. supervised the work of K.Y., I.P. and G.M.

## Supplementary Material

Supplementary Information Supplementary Information

Supplementary Information Geometry, conductance, loading and breaking forces for single-molecule junctions of BT2

Supplementary Information Geometry, conductance, loading and breaking forces for double-molecule junctions of BT2

Supplementary Information Geometry, conductance, loading and breaking forces for single-molecule junctions of NH22

Supplementary Information Geometry, conductance, loading and breaking forces for double-molecule junctions of NH22

Supplementary Information Geometry, conductance, loading and breaking forces for single-molecule junctions of PY2

Supplementary Information Geometry, conductance, loading and breaking forces for double-molecule junctions of PY2

## Figures and Tables

**Figure 1:**
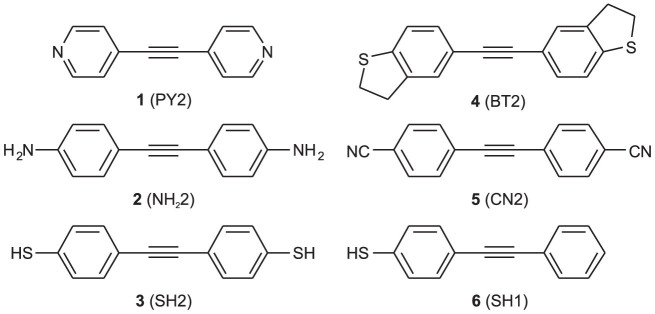
Chemical structures of the 6 compounds studied in this work.

**Table 1:** Experimental maxima of conductance *G_max_*/*G*_0_ and mean breaking force *F_b_*_,*e*_ as well as theoretical mean breaking force *F_b_*_,*t*_ for all studied tolanes in respective conductance ranges

	*G_max_*/*G*_0_	*F_b_*_,*e*_ (nN)	*F_b_*_,*t*_ (nN)
PY2(H)	5 · 10^−4^	1.8 ± 0.7	1.8 ± 0.4
PY2(M)	4 · 10^−5^	1.1 ± 0.5	1.1 ± 0.3
PY2(L)	5 · 10^−6^	0.8 ± 0.4	0.3 ± 0.2
BT2(H)	5 · 10^−3^	0.8 ± 0.3	0.8 ± 0.1
BT2(L)	3 · 10^−4^	0.3 ± 0.2	0.3 ± 0.1
SH2(H)	1.6 · 10^−3^	1.2 ± 0.5	
SH2(L)	2.5 · 10^−4^	0.8 ± 0.4	
SH1(L)	2.5 · 10^−4^	0.6 ± 0.3	
NH_2_2(H)	10^−3^	0.6 ± 0.3	0.7 ± 0.2
NH_2_2(L)	1.8 · 10^−5^	0.6 ± 0.3	0.2 ± 0.1
CN2(H)	8 · 10^−5^	0.5 ± 0.2	

## References

[b1] Li C., Mishchenko A., Pobelov I. & Wandlowski T. Charge transport with single molecules - an electrochemical approach. Chimia 64, 383–390 (2010). 21137713 10.2533/chimia.2010.383

[b2] Aradhya S. V. & Venkataraman L. Single-molecule junctions beyond electronic transport. Nat. Nanotechnol. 8, 399–410 (2013). 23736215 10.1038/nnano.2013.91

[b3] Sun L. *et al.* Single-molecule electronics: from chemical design to functional devices. Chem. Soc. Rev. 43, 7378–7411 (2014). 25099384 10.1039/c4cs00143e

[b4] Grandbois M., Beyer M., Rief M., Clausen-Schaumann H. & Gaub H. E. How strong is a covalent bond? Science 283, 1727–1730 (1999). 10073936 10.1126/science.283.5408.1727

[b5] Bizzarri A. R. & Cannistraro S. Atomic force spectroscopy in biological complex formation: Strategies and perspectives. J. Phys. Chem. B 113, 16449–16464 (2009). 19904973 10.1021/jp902421r

[b6] Noy A. & Friddle R. W. Practical single molecule force spectroscopy: How to determine fundamental thermodynamic parameters of intermolecular bonds with an atomic force microscope. Methods 60, 142–150 (2013). 23531626 10.1016/j.ymeth.2013.03.014

[b7] Choi S. H., Kim B. & Frisbie C. D. Electrical resistance of long conjugated molecular wires. Science 320, 1482–1486 (2008). 18556556 10.1126/science.1156538

[b8] Wang C. *et al.* Oligoyne single molecule wires. J. Am. Chem. Soc. 131, 15647–15654 (2009). 19824644 10.1021/ja9061129

[b9] Kaliginedi V. *et al.* Correlations between molecular structure and single-junction conductance: A case study with oligo(phenylene-ethynylene)-type wires. J. Am. Chem. Soc. 134, 5262–5275 (2012). 22352944 10.1021/ja211555x

[b10] Wang C. *et al.* Electrical characterization of 7 nm long conjugated molecular wires: experimental and theoretical studies. Nanotechnology 18, 044005 (2007).

[b11] Haiss W. *et al.* Variable contact gap single-molecule conductance determination for a series of conjugated molecular bridges. J. Phys.: Condens. Matter 20, 374119 (2008). 21694426 10.1088/0953-8984/20/37/374119

[b12] Hong W. *et al.* An MCBJ case study: The influence of pi-conjugation on the single-molecule conductance at a solid/liquid interface. Beilstein J. Nanotechnol. 2, 699–713 (2011). 22043460 10.3762/bjnano.2.76 PMC3201624

[b13] Park Y. S. *et al.* Frustrated rotations in single-molecule junctions. J. Am. Chem. Soc. 131, 10820–10821 (2009). 19722660 10.1021/ja903731m

[b14] Mishchenko A. *et al.* Influence of conformation on conductance of biphenyl-dithiol single-molecule contacts. Nano Lett. 10, 156–163 (2010). 20025266 10.1021/nl903084b

[b15] Finch C. M. *et al.* Conformation dependence of molecular conductance: chemistry versus geometry. J. Phys.: Condens. Matter 20, 022203 (2008).

[b16] Ashwell G. J. *et al.* Molecular bridging of silicon nanogaps. ACS Nano 4, 7401–7406 (2010). 21082817 10.1021/nn102460z

[b17] Ashwell G. J. *et al.* Synthesis of covalently linked molecular bridges between silicon electrodes in CMOS-based arrays of vertical Si/SiO_2_/Si nanogaps. Angew. Chem. Int. Ed. 50, 8722–8726 (2011). 10.1002/anie.201102791 21793136

[b18] Chen F., Li X., Hihath J., Huang Z. & Tao N. Effect of anchoring groups on single-molecule conductance: Comparative study of thiol-, amine-, and carboxylic-acid-terminated molecules. J. Am. Chem. Soc. 128, 15874–15881 (2006). 17147400 10.1021/ja065864k

[b19] Li C. *et al.* Charge transport in single Au|alkanedithiol|Au junctions: Coordination geometries and conformational degrees of freedom. J. Am. Chem. Soc. 130, 318–326 (2008). 18076172 10.1021/ja0762386

[b20] Hong W. *et al.* Single molecular conductance of tolanes: Experimental and theoretical study on the junction evolution dependent on the anchoring group. J. Am. Chem. Soc. 134, 2292–2304 (2012). 22175273 10.1021/ja209844r

[b21] Hong W. *et al.* Trimethylsilyl-terminated oligo(phenylene ethynylene)s: An approach to single-molecule junctions with covalent Au-C *σ*-bonds. J. Am. Chem. Soc. 134, 19425–19431 (2012). 23126569 10.1021/ja307544w

[b22] Moreno-García P. *et al.* Single-molecule conductance of functionalized oligoynes: Length dependence and junction evolution. J. Am. Chem. Soc. 135, 12228–12240 (2013). 23875671 10.1021/ja4015293

[b23] Kaliginedi V. *et al.* Promising anchoring groups for single-molecule conductance measurements. Phys. Chem. Chem. Phys. 16, 23529–23539 (2014). 25285778 10.1039/c4cp03605k

[b24] Florin E. L., Moy V. T. & Gaub H. E. Adhesion forces between individual ligand-receptor pairs. Science 264, 415–417 (1994). 8153628 10.1126/science.8153628

[b25] Lee G. U., Chrisey L. A. & Colton R. J. Direct measurement of the forces between complementary strands of DNA. Science 266, 771–773 (1994). 7973628 10.1126/science.7973628

[b26] Rief M., Gautel M., Oesterhelt F., Fernandez J. M. & Gaub H. E. Reversible unfolding of individual titin immunoglobulin domains by AFM. Science 276, 1109–1112 (1997). 9148804 10.1126/science.276.5315.1109

[b27] Strunz T., Oroszlan K., Schäfer R. & Güntherodt H.-J. Dynamic force spectroscopy of single DNA molecules. Proc. Natl. Acad. Sci. U. S. A. 96, 11277–11282 (1999). 10500167 10.1073/pnas.96.20.11277 PMC18024

[b28] Marszalek P. E. *et al.* Mechanical unfolding intermediates in titin modules. Nature 402, 100–103 (1999). 10573426 10.1038/47083

[b29] Xu B., Xiao X. & Tao N. J. Measurements of single-molecule electromechanical properties. J. Am. Chem. Soc. 125, 16164–16165 (2003). 14692738 10.1021/ja038949j

[b30] Frei M., Aradhya S. V., Koentopp M., Hybertsen M. S. & Venkataraman L. Mechanics and chemistry: single molecule bond rupture forces correlate with molecular backbone structure. Nano Lett. 11, 1518–1523 (2011). 21366230 10.1021/nl1042903

[b31] Nef C., Frederix P. L. T. M., Brunner J., Schönenberger C. & Calame M. Force-conductance correlation in individual molecular junctions. Nanotechnology 23, 365201 (2012). 22909952 10.1088/0957-4484/23/36/365201

[b32] Meisner J. S. *et al.* Importance of direct metal-*π* coupling in electronic transport through conjugated single-molecule junctions. J. Am. Chem. Soc. 134, 20440–20445 (2012). 23167533 10.1021/ja308626m

[b33] Pobelov I. V. *et al.* An approach to measure electromechanical properties of atomic and molecular junctions. J. Phys.: Condens. Matter 24, 164210 (2012). 22466399 10.1088/0953-8984/24/16/164210

[b34] Aradhya S. V., Frei M., Hybertsen M. S. & Venkataraman L. Van der Waals interactions at metal/organic interfaces at the single-molecule level. Nat. Mater. 11, 872–876 (2012). 22886066 10.1038/nmat3403

[b35] Frei M., Aradhya S. V., Hybertsen M. S. & Venkataraman L. Linker dependent bond rupture force measurements in single-molecule junctions. J. Am. Chem. Soc. 134, 4003–4006 (2012). 22338625 10.1021/ja211590d

[b36] Chen I.-W. P. *et al.* Tactile-feedback stabilized molecular junctions for the measurement of molecular conductance. Angew. Chem., Int. Ed. 52, 2449–2453 (2013). 10.1002/anie.201207116 23341350

[b37] Wu S. *et al.* Molecular junctions based on aromatic coupling. Nat. Nanotechnol. 3, 569–574 (2008). 18772920 10.1038/nnano.2008.237

[b38] Martín S. *et al.* Identifying diversity in nanoscale electrical break junctions. J. Am. Chem. Soc. 132, 9157–9164 (2010). 20536142 10.1021/ja103327f

[b39] He J., Lin L., Zhang P. & Lindsay S. Identification of DNA basepairing via tunnel-current decay. Nano Lett. 7, 3854–3858 (2007). 18041859 10.1021/nl0726205 PMC2311509

[b40] Kolivoška V. *et al.* Electrochemical control of a non-covalent binding between ferrocene and beta-cyclodextrin. Chem. Commun. 50, 11757–11759 (2014). 10.1039/c4cc04102j 25144878

[b41] Pobelov I. V. *et al.* Electrochemical current-sensing atomic force microscopy in conductive solutions. Nanotechnology 24, 115501 (2013). 23448801 10.1088/0957-4484/24/11/115501

[b42] Mohos M. *et al.* Breaking force and conductance of gold nanojunctions: Effect of humidity. J. Phys. Chem. Lett. 5, 3560–3564 (2014). 10.1021/jz5019459 26278610

[b43] Jones L. II, Schumm J. S. & Tour J. M. Rapid solution and solid phase syntheses of oligo(1,4-phenylene ethynylene)s with thioester termini: Molecular scale wires with alligator clips. derivation of iterative reaction efficiencies on a polymer support. J. Org. Chem. 62, 1388–1410 (1997).

[b44] Soler J. M. *et al.* The SIESTA method for ab initio order-N materials simulation. J. Phys.: Condens. Matter 14, 2745–2779 (2002).

[b45] Rocha A. R. *et al.* Towards molecular spintronics. Nat. Mater. 4, 335–339 (2005). 15750597 10.1038/nmat1349

[b46] Rocha A. R. *et al.* Spin and molecular electronics in atomically generated orbital landscapes. Phys. Rev. B 73, 085414 (2006).

