# Supplementary material for: Correlation of breaking forces, conductances and geometries of molecular junctions
Source: Sci Rep. 2015 Mar 11;5:9002. doi: 10.1038/srep09002 (PMC4355744; doi:10.1038/srep09002)
Supplement: Supplementary Information [file srep09002-s1.pdf]

Supplementary Information: Correlation of breaking forces, conductances and geometries of molecular junctions

Koji Yoshida[1], Ilya V. Pobelov[1,*], David Zsolt Manrique[2], Thomas Pope[2], Gábor Mészáros[1,3], Murat Gulcur[4], Martin R. Bryce[4,*], Colin J. Lambert[2,*], Thomas Wandlowski[1]

[1] Department of Chemistry and Biochemistry, University of Bern, Freiestrasse 3, 3012 Bern, Switzerland. [2] Department of Physics, Lancaster University, Lancaster LA1 4YB, United Kingdom. [3] Research Centre for Natural Sciences, HAS, Magyar tudósok krt. 2, H-1117 Budapest, Hungary. [4] Department of Chemistry, Durham University, South Road, Durham, DH1 3LE, United Kingdom.

E-mail: ilya.pobelov@iac.unibe.ch, m.r.bryce@durham.ac.uk, c.lambert@lancaster.ac.uk

Supplementary Figures

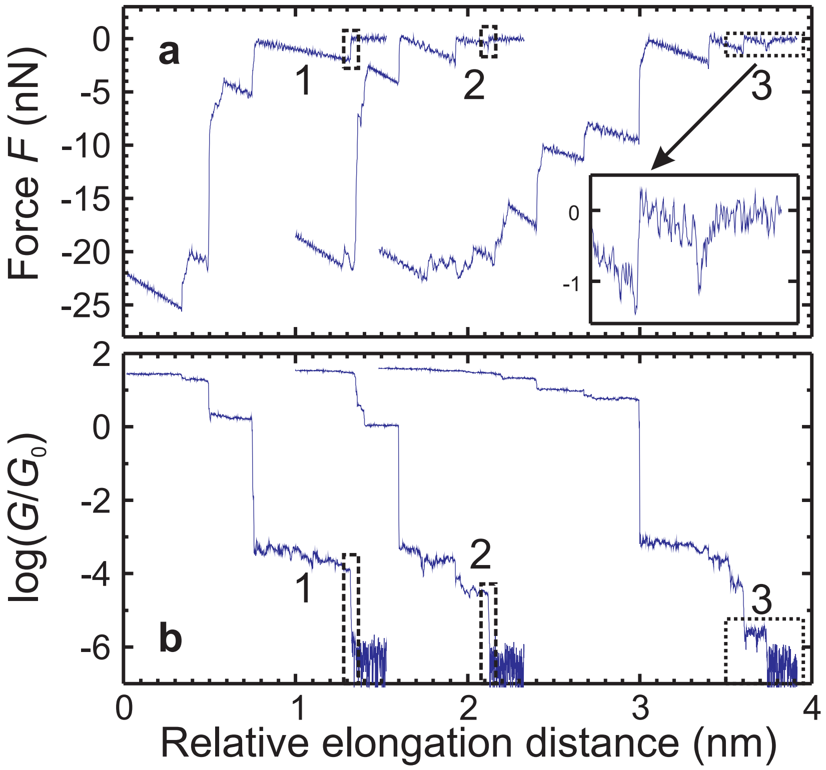

Supplementary Figure 1: Typical experimental elongation traces measured by CSAFM in solutions of PY2 show the evolution of the force F applied to the junction (a) and of the logarithm of normalised junction conductance $\log(G/G_0)$ (b) as a function of the relative elongation distance. The traces show breaking of the molecular junctions with conductances in the high (1), medium (2) and low (3) range (see below). The dashed rectangles mark breaking regions in traces 1 and 2. Inset in panel (a) shows the part of force trace 3 marked by a dotted rectangle. The corresponding part of the conductance trace is marked by a dotted rectangle in panel (b).

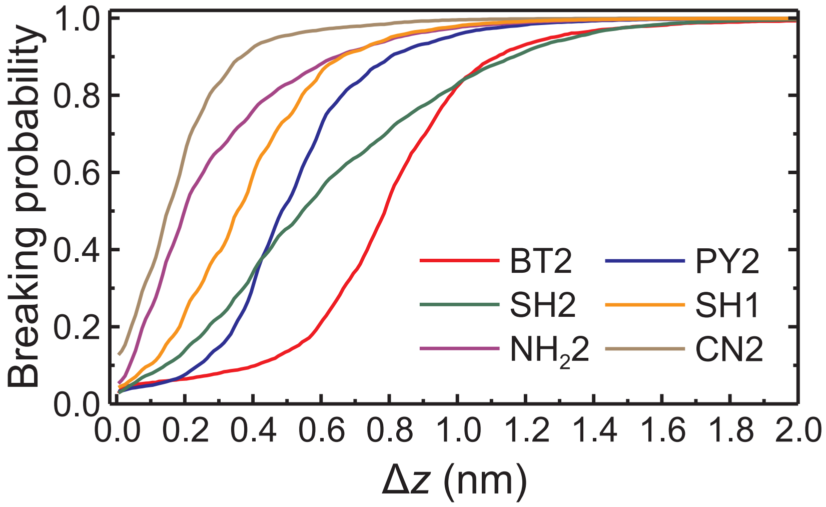

Supplementary Figure 2: Statistical probability of the junction breaking before its extension to the relative distance Δz for all studied tolanes.

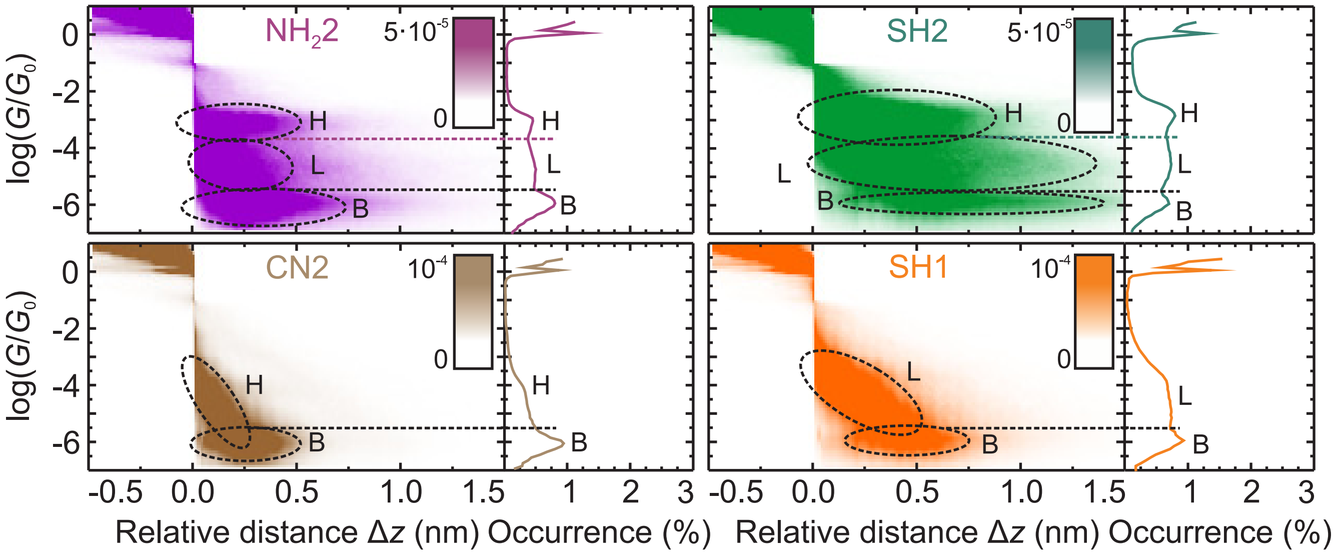

Supplementary Figure 3: 2D histograms of $\log(G/G_0)$ versus Δz(left) and 1D histograms of $\log(G/G_0)$ (right) constructed from all data points of the measured elongation traces for the molecular junctions formed by $NH_2$2, CN2, SH2 and SH1. The occurrence of data points in the 2D (size 0.01 nm×0.1) and 1D (size 0.1) bins is given by the colour scale and by the solid lines, respectively. The dashed ellipses in the 2D histograms and letters in 1D histograms mark features of molecular junctions with high (H) and low (L) conductance as well as features of the baseline conductance (B). The latter are separated from the molecule-specific features by a dashed black horizontal line. The coloured dashed horizontal lines correspond to the positions of borders between the conductance ranges $\log(G_{HL}/G_0)$ (Table 2).

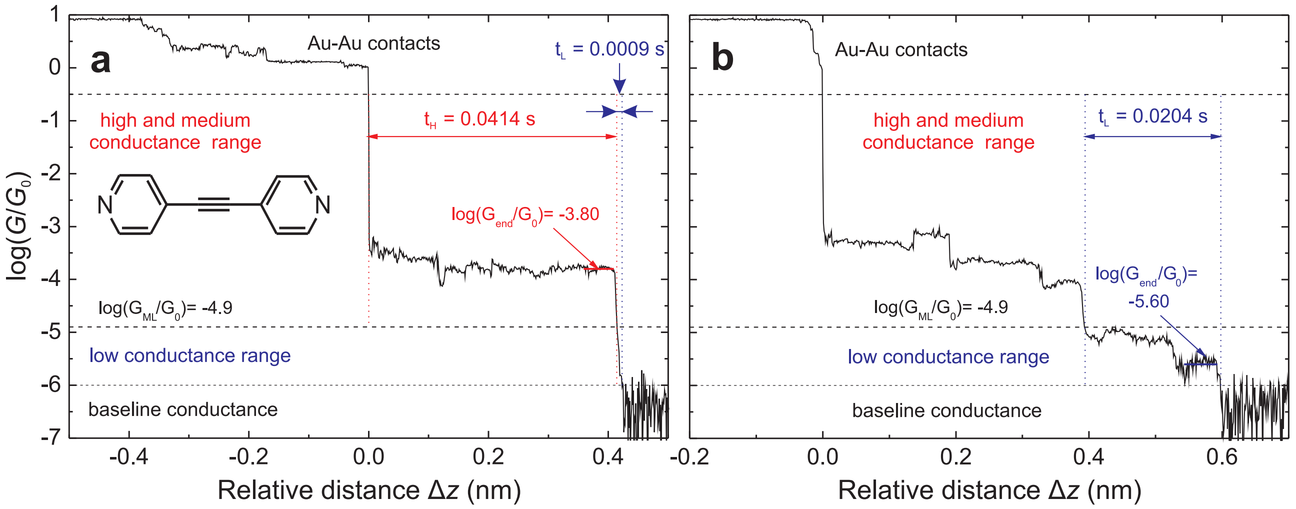

Supplementary Figure 4: An illustration of the procedure employed to select traces for the force analysis and to calculate the conductance at the end of the junction extension. The presented traces were measured for PY2 junctions and exhibit conductance plateaus in the high (a) and the low (b) conductance range.

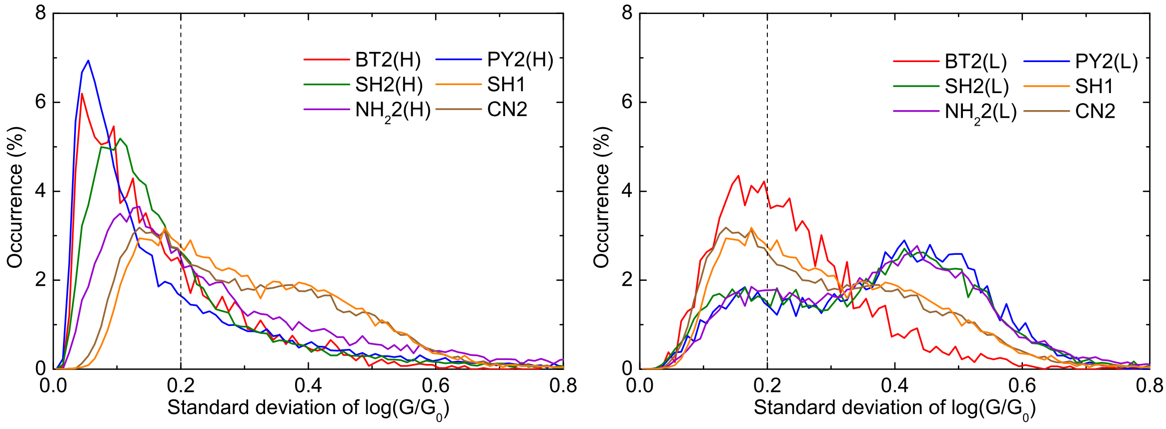

Supplementary Figure 5: Distributions of σ_{end} calculated from all traces with a sufficiently long ($t_{H,L} > 0.01$ s) conductance plateau in the respective conductance range. Bin size of 0.01 was employed. The distributions are normalised by the total amount of traces with sufficiently long conductance plateaus in each range. The distributions for SH1 and CN2 are plotted in both panels. The vertical dashed line indicates an applied cut-off limit of σ_{end} equal to 0.2.

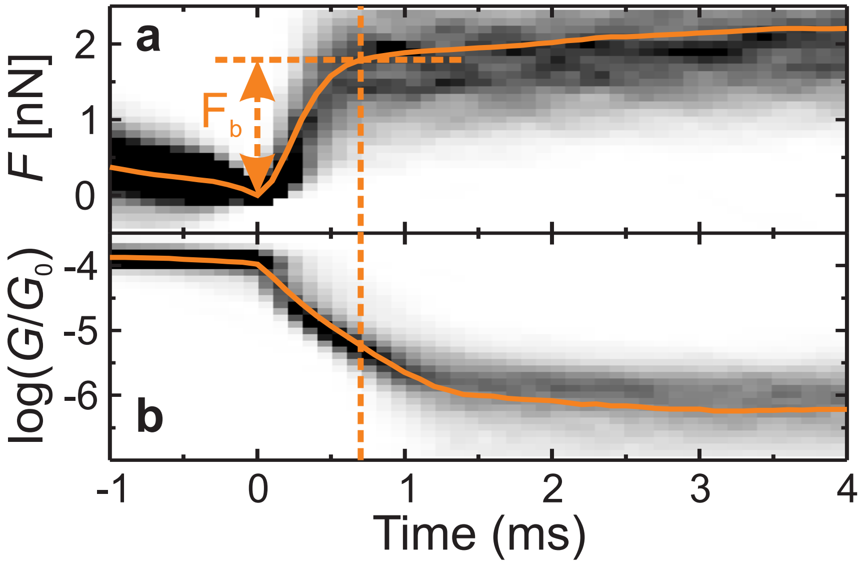

Supplementary Figure 6: 2D force (a) and $\log(G/G_0)$ (b) histograms constructed from 694 realigned traces for PY2 junctions with $-3.9 < \log(G_{end}/G_0) < -3.8$. Bin sizes are 0.1 ms×0.1 nN (a) and 0.1 ms×0.1 (b). The solid curves are mean force and $\log(G/G_0)$ traces calculated for the given data set. The dashed vertical line indicates a breaking time of 0.7 ms.

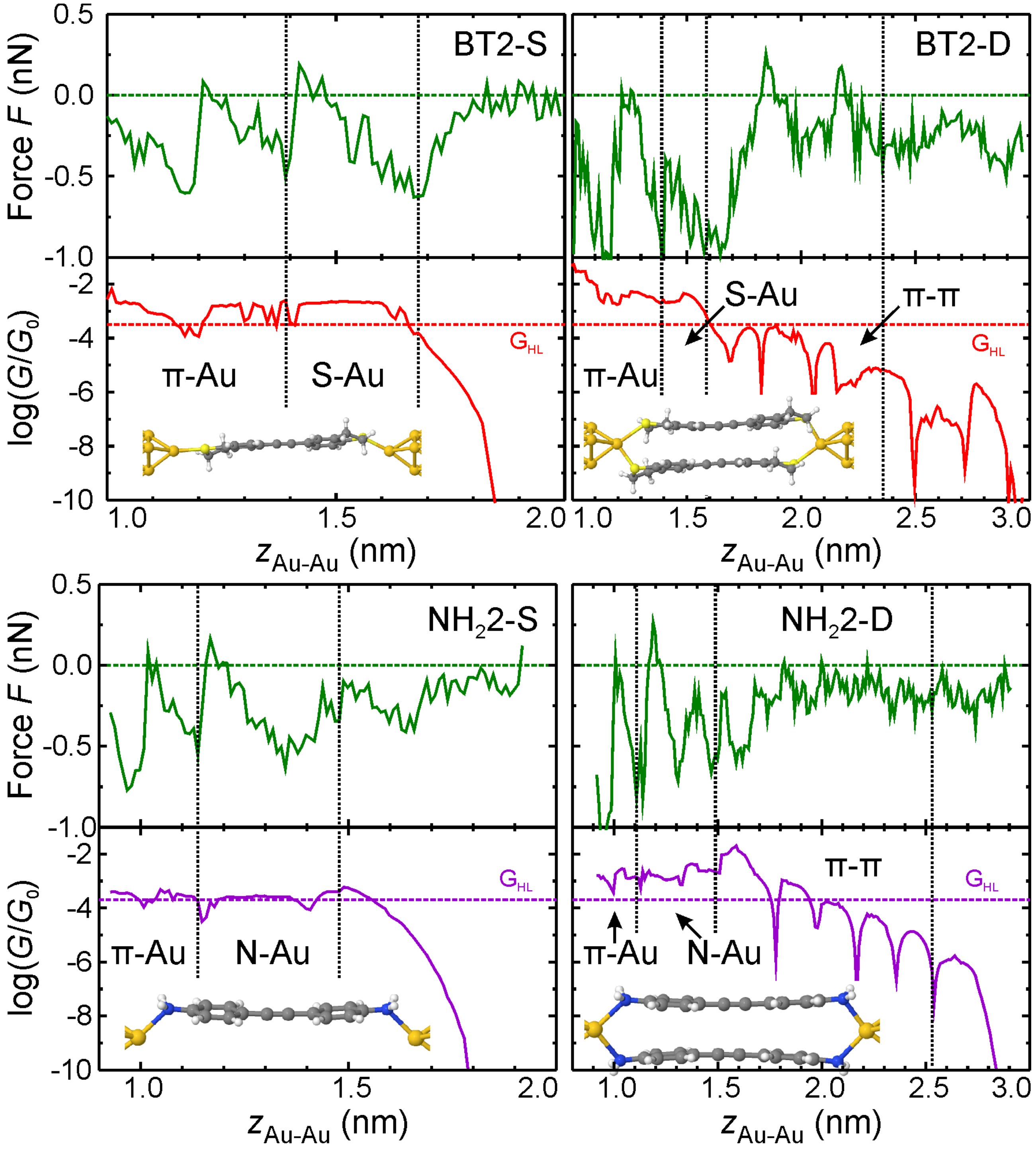

Supplementary Figure 7: Theoretical loading force F and logarithm of the normalised conductance $\log(G/G_0)$ of single (left) and double (right) molecule junctions of BT2 and $NH_2$2 as a function of the electrode separation z_{Au-Au}. The vertical dashed lines correspond to the transitions between configurations with different type of coupling. The insets show geometry of covalent junctions just before breaking. The horizontal dashed lines in conductance panels indicate the position of experimental border between the high and the low conductance ranges G_{HL}.

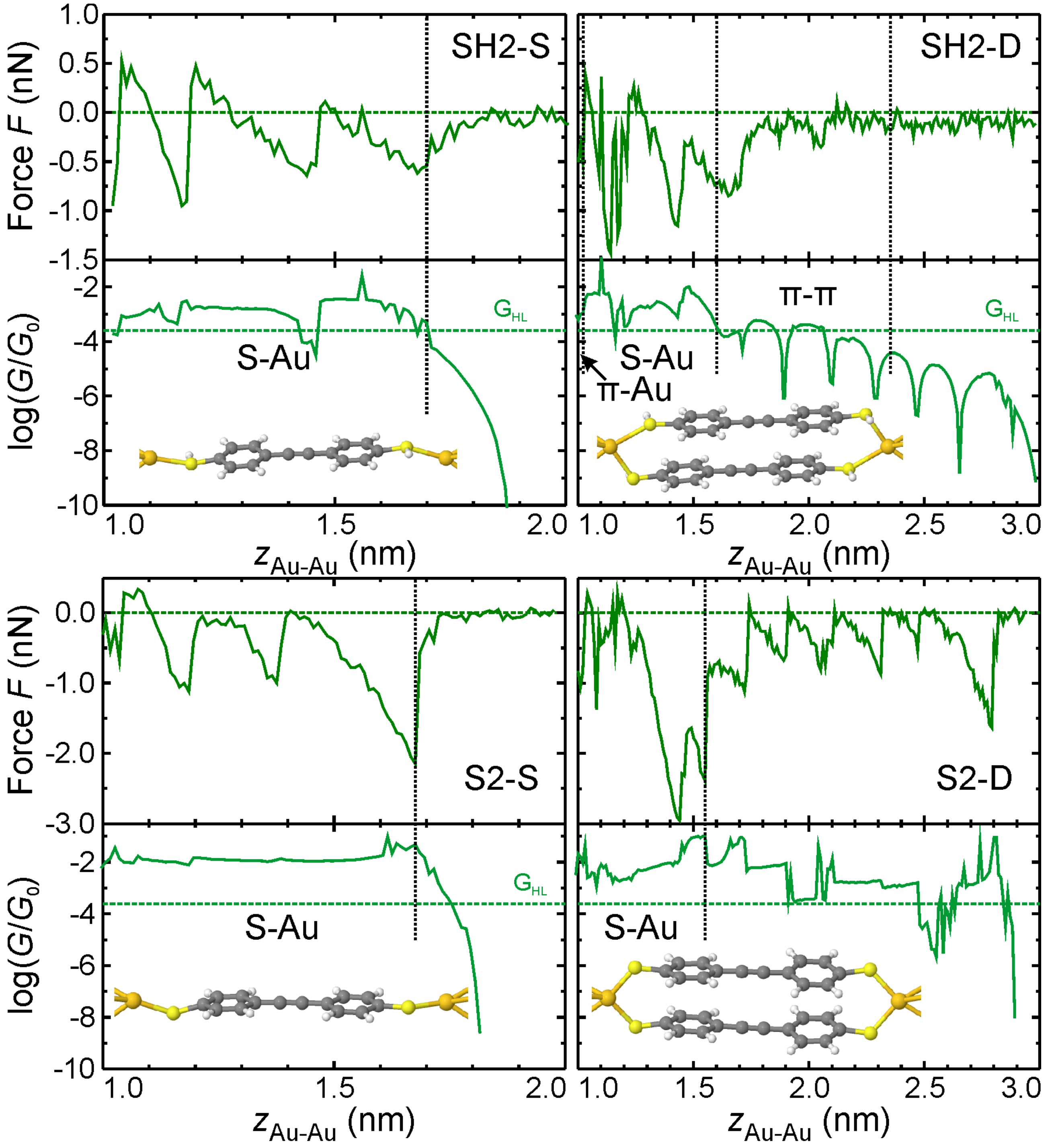

Supplementary Figure 8: Theoretical loading force F and logarithm of the normalised conductance $\log(G/G_0)$ of single (left) and double (right) molecule junctions of SH2 and S2 as a function of the electrode separation z_{Au-Au}. The vertical dashed lines correspond to the transitions between configurations with different type of coupling. The insets show geometry of covalent junctions just before breaking. The horizontal dashed lines in conductance panels indicate the position of experimental border between the high and the low conductance ranges G_{HL}. We note that S2 is a free radical. After detachment of one of the anchoring groups in both molecules of the double-molecule junction (vertical dashed lines in 8, panel S2-D), S2 molecules form covalent dimers instead of π-π stacked dimers.

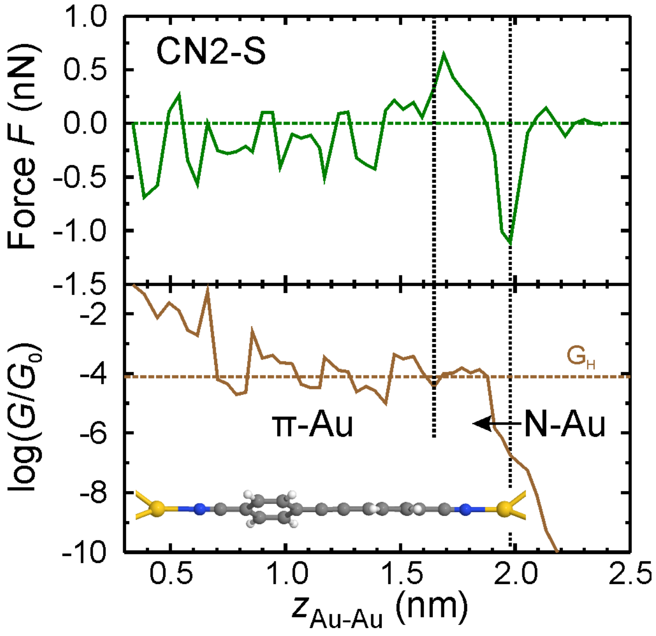

Supplementary Figure 9: Theoretical loading force F and logarithm of the normalised conductance $\log(G/G_0)$ of a junction with single CN2 molecule as a function of the electrode separation z_{Au-Au}. The vertical dashed lines correspond to the transitions between configurations with π-Au and N-Au couplings, and junction breaking. The inset shows the geometry of the junction just before breaking. The horizontal dashed lines in the conductance panels indicate the position of experimental conductance G_H.

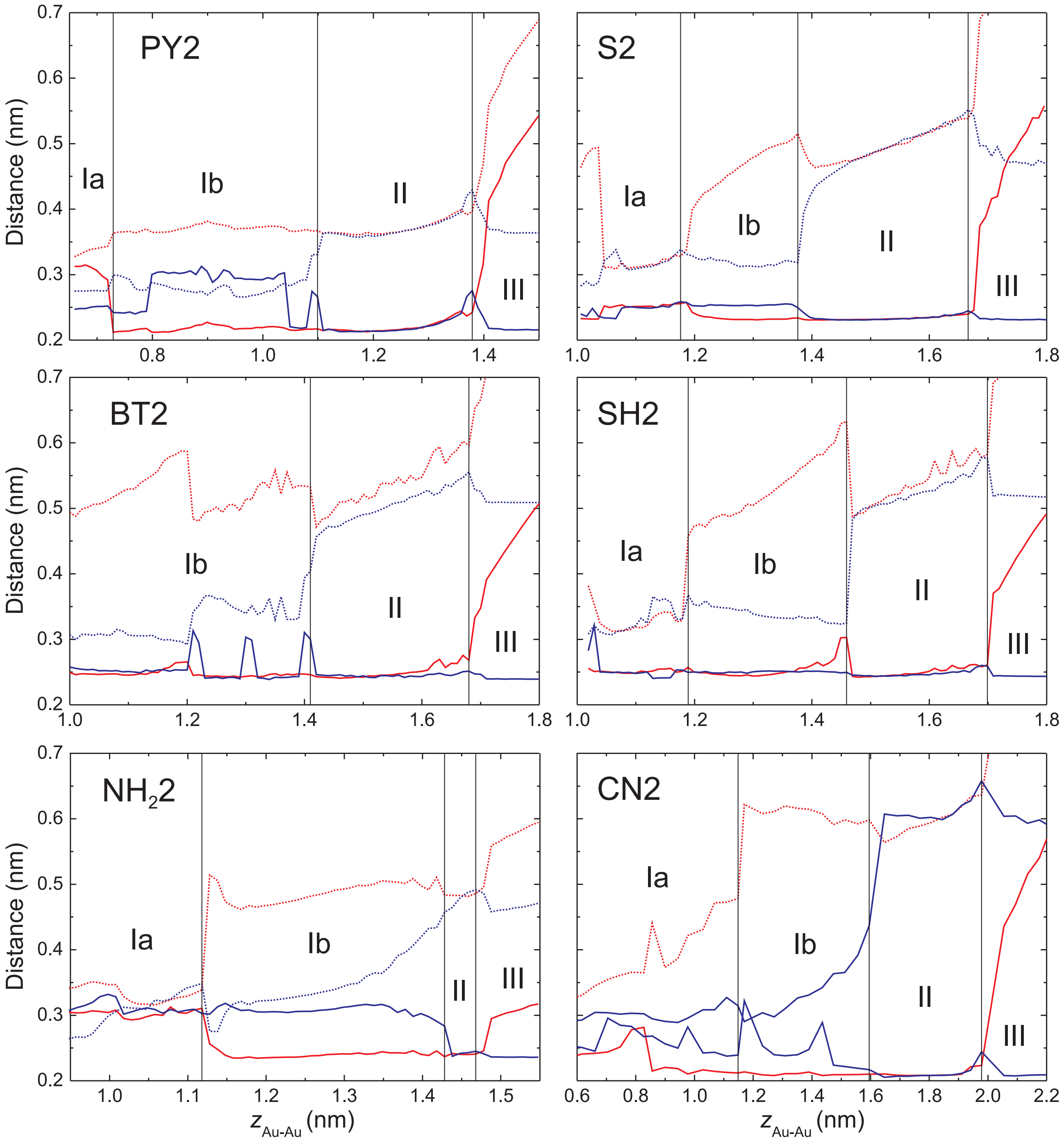

Supplementary Figure 10: $d_{Anchor-Au}$ (solid) and $d_{Ring-Au}$ (dotted) determined for upper (red) and lower (blue) pyramid in single-molecule junctions. The vertical black lines mark transitions between different geometric stages.

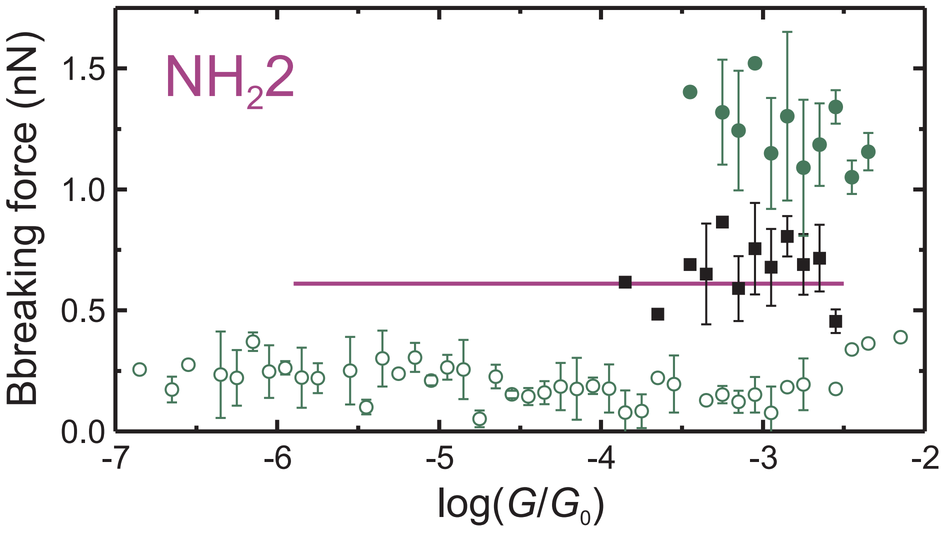

Supplementary Figure 11: Mean theoretical breaking force $F_{b,t}$ for simulated single-molecule (squares) and its standard deviation (error bar) and double-molecule (circles) junctions of $NH_2$2. Full and open circles indicate covalent and $\pi - \pi$ stacked dimer junctions formed by two molecules. The solid purple line represents the mean value of the experimental breaking force in the corresponding range of G_{end} as shown in Figure 5.

Supplementary Tables

Supplementary Table 1: Junction formation probability determined in STMBJ/MCBJ experiments (Ref. 1) compared to the median of L_{exp}.

	BT2	PY2	SH2	SH1	NH_22	CN2
Median of L_{exp} (nm)	0.79	0.49	0.55	0.35	0.20	0.15
Junction formation probability (%) (Ref. 1)	100	100	90	–	60	63

Supplementary Table 2: Positions of the conductance peaks and of the borders between the conductance ranges as determined from 1D and 2D histograms of $\log(G/G_0)$ (Fig. 1 and Supplementary Fig. 3) for the studied tolanes.

	PY2	BT2	NH_22	CN2	SH2	SH1
$\log(G_H/G_0)$	-3.3	-2.3	-3.0	-4.1^a	-2.8	
$\log(G_{HM}/G_0)$	-3.9					
$\log(G_M/G_0)$	-4.4^a					
$\log(G_{ML}/G_0)$	-4.9					
$\log(G_{HL}/G_0)$		-3.5	-3.7		-3.6	
$\log(G_L/G_0)$	-5.3^a	-4.7	-4.75		-4.5^b	-4.5^b

^{a} Approximately determined from a shoulder. ^{b} Approximately determined from a broad peak.

Supplementary Table 3: Conductance of studied tolanes as quoted in the literature. The comparison of results obtained in the CSAFM experiment in this work with those obtained in previous STMBJ and MCBJ experiments demonstrates a very good agreement with the data obtained in our group employing two different techniques (Refs. 1, 2) and a fairly good agreement with the results of other groups.

	PY2	BT2	$NH_2$2	CN2	SH2	SH1
	STMBJ, Ref. 1					
$\log(G_H/G_0)$<br>$\log(G_M/G_0)$<br>$\log(G_L/G_0)$	−3.4<br>−4.1<br>−5.8	−2.5<br><br>−5.1	−3.2<br><br>−4.8	−4.8	−2.8<br><br>−4.6	
	MCBJ, Ref. 1					
$\log(G_H/G_0)$<br>$\log(G_L/G_0)$	−3.3<br>−5.8	−2.5<br>−4.9	−3.2<br>−4.8	−4.7	−2.7<br>−4.7	
	STMBJ, Ref. 2					
$\log(G_H/G_0)$<br>$\log(G_M/G_0)$<br>$\log(G_L/G_0)$	−3.3<br>−4.5<br>−6.0		−3.1<br><br>−4.8	−4.6	−2.7<br><br>−4.7	
	MCBJ, Ref. 2					
$\log(G_H/G_0)$<br>$\log(G_L/G_0)$	−3.3<br>−6.0		−3.1<br>−4.9	−4.6	−2.7<br>−4.2/−5.8	
	STMBJ, Ref. 3					
$\log(G_A/G_0)$<br>$\log(G_B/G_0)$<br>$\log(G_C/G_0)$	−4.05<br>−3.46<br>−2.54					
	STMBJ, Ref. 4					
$\log(G_H/G_0)$<br>$\log(G_L/G_0)$	−3.7<br>−4.3					
	STMBJ, Ref. 5					
$\log(G/G_0)$			-3.25			
	STMBJ, Ref. 6					
$\log(G/G_0)$			-3.25			
	STMBJ, Ref. 7					
$\log(G/G_0)$					-3.6	
	MCBJ, Ref. 8					
$\log(G_L/G_0)$						−4.1

Supplementary Table 4: Geometrical parameters of the simulated molecules.

Molecule	PY2	BT2	$NH_2$2	SH2	CN2
L (nm)	0.98	1.31	1.26	1.31	1.49
L_{ar} (nm)	0.98	0.97	0.98	0.98	0.98
$d_{Anchor-Au}$ (nm)	0.21	0.24	0.24	0.23	0.21

Supplementary Table 5: Average $d_{Anchor-Au}$ and $d_{Ring-Au}$ distances for bound aromatic rings during the stage Ib of junction elongation and their difference.

Molecule	PY2	BT2	$NH_2$2	SH2	S2	CN2
ring-gold distance (nm)	0.283	0.329	0.488	0.339	0.319	0.342
anchor-gold distance (nm)	0.279	0.256	0.240	0.250	0.253	0.247
difference (nm)	0.004	0.073	0.248	0.089	0.066	0.095

Supplementary Table 6: Theoretical mean breaking forces $F_{b,t}$ determined for tolanes in different coupling arrangements in respective conductance ranges.

Junction and coupling type	max $\log(G/G_0)$	min $\log(G/G_0)$	$F_{b,t}$ (nN)	standard deviation (nN)
PY2-S, π-Au	−2.7	−4.2	1.13	0.31
PY2-S, N-Au	−3.6	−6.3	1.05	0.26
PY2-S, all	−2.7	−6.3	1.10	0.29
PY2-D, π-Au	−2.5	−3.4	1.79	0.41
PY2-D, N-Au	−3.6	−4.7	1.71	0.31
PY2-D, π-π	−5.0	−7.2	0.34	0.18
BT2-S	−4.0	−2.2	0.80	0.12
BT2-D, π-Au and S-Au	−1.3	−3.0	1.49	0.36
BT2-D, π-π	−3.2	−10.2	0.25	0.10
$NH_2$2-S	−2.6	−3.9	0.69	0.15
$NH_2$2-D, π-Au and N-Au	−2.3	−3.4	1.21	0.25
$NH_2$2-D, π-π	−2.1	−7.6	0.19	0.10

Supplementary Table 7: Experimental mean breaking forces $F_{b,e}$ assigned to the single-molecule junctions; terminal breaking forces $F_{t,t}$ estimated from the simulated elongation traces and theoretical breaking forces $F_{b,t}$ for single-molecule junctions.

	BT2	PY2	SH2		$NH_2$2	CN2
$F_{b,e}$ (nN)	0.8	1.1	1.2		0.6	0.5
	BT2	PY2	SH2	S2	$NH_2$2	CN2
$F_{t,t}$ (nN)	0.6	1.4	0.6	2.1	0.34	1.1
$F_{b,t}$ (nN)	0.8	1.1			0.7	

Supplementary Table 8: Experimental mean breaking forces $F_{b,e}$ assigned to the π-π stacked dimer junctions; terminal breaking forces $F_{t,t}$ estimated from the simulated elongation traces and theoretical breaking forces $F_{b,t}$ for double-molecules junction. To estimate the energy of binding between two molecules, we relaxed the geometry of a double molecule junction such as D4 (Figure 3) in the absence of the gold pyramids.The binding energy E_{bind} was then calculated between the two molecules in the optimised geometry using the counter-poise method combined with SIESTA.

	BT2	PY2	SH2		$NH_2$2	SH1
$F_{b,e}$ (nN)	0.34	0.8	0.82		0.63	0.55
	BT2	PY2	SH2	S2	$NH_2$2	
$F_{t,t}$ (nN)	0.35	0.28	0.18		0.28	
$F_{b,t}$ (nN)	0.25	0.34			0.19	
E_{bind} (eV)	0.29	0.43	0.31		0.39	

Supplementary Notes

Supplementary Note 1. Individual CSAFM traces

Supplementary Figure 1 displays few examples of individual elongation traces of PY2 junctions, illustrating the simultaneous evolution of junction conductance G and force F acting on the cantilever. The original time scale was transformed into a distance scale by multiplication with the pulling rate r. The traces show plateaus at $\log(G/G_0) \geq 0$, which correspond to the formation and the subsequent breaking of Au-Au contacts[9]. Plateaus with $\log(G/G_0) < 0$ represent the formation of molecular junctions. Irreversible inelastic deformations or the breaking of junctions are seen as sharp conductance drops[1,2] accompanied by sudden force jumps. The latter are caused by junction relaxations[9]. A "baseline" conductance with $\log(G/G_0) \leq -6$, as determined by the sensitivity of the employed current amplifier, is measured in the absence of an electrical contact between the two gold electrodes, i.e. when the junction is completely broken.

Supplementary Note 2. Stability of the molecular junctions

To compare stability of molecular junctions formed by different tolanes, we analysed extension of junctions before breaking. An experimental extension length of a molecular junction L_{exp} was defined as a position of the first data point with conductance $G < 10^{-6}\ G_0$, i.e. below the baseline level, with respect to the breaking of gold-gold contact (Δz scale as introduced in the main text). Then, we calculated for each tolane the number of traces with $L_{exp} < \Delta z$ as a function of Δz, and normalised it by the number of measured traces. The obtained dependences (Supplementary Fig. 2) represent the statistical probability of the junction breaking before the extension to the given distance Δz and therefore characterise the stability of molecular junctions formed by studied tolanes. The medians of L_{exp}, corresponding to the Δz at which half of the traces are already broken, are listed in Supplementary Table 1.

The difference between the tolanes is drastic. BT2 forms the most stable junctions. The S-like shape of the BT2 curve in Supplementary Fig. 2 indicates that few traces are broken at low extensions, e.g. only 20% at $\Delta z = 0.6$ nm, and the median of L_{exp} equal to 0.8 nm is the highest one. The shape of the breaking probability curve for PY2 resembles that of BT2 shifted towards lower Δz. For the purpose of discussion, we classify BT2 and PY2 as tolanes with the high probability of the junction

formation. CN2, $NH_2$2 and SH1, on the other hand, show a significant number of junctions already broken at low Δz. The shapes of the breaking probability curves are rather Langmuir-like than S-shaped, with the small slowly raising part at low Δz for SH1. We classify CN2, $NH_2$2 and SH1 as tolanes with the low probability of junction formation. SH2 represents an intermediate case. On one hand, the shape of the breaking probability curve for SH2 is rather Langmuir-like. On the other hand, the slope of its linear part is lower than for other tolanes, indicating broader distribution over L_{exp}, and for high Δz it overlaps with the curve for BT2. We attribute the shape of the SH2 curve to the large variation of possible binding geometries of thiol groups to gold electrodes[10].

We note that in Ref. 1 the stability of molecular junctions was characterised by a probability of junction formation. To determine the latter, we constructed histograms of characteristic plateau length, i.e. a position of the last point of conductance plateau in Δz scale. If only one peak of plateau length was observed, we implied the junction formation probability of 100%. If two peaks of the characteristic length were found, we attributed the peaks at lower and higher lengths to the formation of junctions without and with molecules, respectively. By comparing the area of the two peaks, as obtained by fitting the distribution with two Gaussian peaks, we estimated the junction formation probability. The trend of junction stability found in this work agrees well with the one reported in Ref. 1 (Supplementary Table 1).

Supplementary Note 3. Experimental breaking forces

Experimental breaking forces $F_{b,e}$ as a function of junction conductance before its breaking were evaluated according to the following procedure. First, we calculated for each individual trace the time (or the equivalent moving distance of the probe) required for the junction to travel through a specific conductance range. An example for two conductance traces measured for PY2 junctions is shown in Supplementary Fig. 4; the medium conductance range of PY2 was treated together with the high conductance range. In case of junctions exhibiting two conductance ranges (PY2, BT2, $NH_2$2, SH2) we analysed the travel time from $\log(G/G_0) = -0.5$ to $\log(G_{HL}/G_0)$ ($\log(G_{ML}/G_0)$ for PY2) t_H(Supplementary Fig. 4a); and from $\log(G_{HL}/G_0)$ ($\log(G_{ML}/G_0)$ for PY2) to $\log(G/G_0) = -6$ for the low conductance range t_L (Supplementary Fig. 4b). For junctions displaying only one conductance range (CN2, SH1) we analysed the travel time from $\log(G/G_0) = -0.5$ to $\log(G/G_0) = -6$. We considered 0.01 s (0.1 nm) as the lower limit of travel time (distance) indicating the formation of a

molecular junction. As we are interested in junction breaking events, we only considered traces in the high conductance range that travel through the lower conductance range in less than 0.003 s (0.03 nm), and all traces that show a junction formation in the low conductance range. Then, we calculated for $\log(G/G_0)$ the mean value (further taken as $\log(G_{end}/G_0)$) and the standard deviation σ_{end} in the range from 5.5 ms (0.055 nm) to 0.5 ms (0.005 nm) before the junction breaking. The latter is indicated by the transition between the high (or medium) and low conductance at G_{HL} (G_{ML} for PY2) for the high conductance range (Supplementary Fig. 4a) or the transition from the low conductance range to baseline level set at 10^{-6} G_0 (Supplementary Fig. 4b). In case of a single conductance range, the baseline limits were applied. An offset of 0.5 ms was used to skip data points before the moment of the actual junction breaking event and its detection. 0.5 ms is a typical apparent time of junction breaking in our experiments (see below).

The standard deviation of $\log(G/G_0)$ at the end of a conductance plateau, σ_{end}, reflects the variation of conductance and thus the stability of an individual molecular junction formed. We compared the stability of molecular junctions formed by the studied tolanes by constructing the distributions of σ_{end} determined for all traces with sufficiently long ($t_{H,L} > 0.01$ s) conductance plateau in the respective conductance range (Supplementary Fig. 5), as obtained before application of further selection criteria. The number of low-noise junctions obtained in the high-conductance range correlate well with the junction formation probability (Supplementary Table 1). In the low-conductance range the lowest noise is found for BT2, while the σ_{end} distribution for PY2, SH2 and $NH_2$2 appears to be very similar. We found that traces with $\sigma_{end} > 0.2$ appear too noisy and do not represent stable junctions before a breaking event. Therefore, we removed them from the further analysis.

The traces selected as described above were grouped according to $\log(G_{end}/G_0)$ in 0.1-wide bins. The traces of one group were realigned by taking the last point before the breaking event as zero in new scales of time and force. The breaking event was determined by a decrease in conductance below the breaking limit, which was typically set 0.2 units below the lower limit of $\log(G_{end}/G_0)$ for a given group (e.g. at $\log(G/G_0) = -4.1$ for data in Supplementary Fig. 6). Then, the time scale was binned with a step of 0.1 ms, and the mean value as well as the standard deviation of force and $\log(G/G_0)$ values from all traces falling into a given time bin were calculated.

The obtained dependences represent the evolution of the force and conductance after breaking of molecular junctions with conductance in a certain range. As an example, Supplementary Fig.

6 illustrates the 2D histograms from all aligned traces of PY2 with the conductance in the range $-3.9 < \log(G_{end}/G_0) < -3.8$, which corresponds to data in the high (H) conductance range. The traces show a rapid increase of the force, followed by a long interval of constant or slowly varying force. The initial force increase typically lasts 0.5 to 0.7 ms after breaking the junction. This finite breaking time is due to the limited bandwidth of the experimental setup. The force value attained after the initial breaking time and its standard deviation were assigned to the mean breaking force $F_{b,e}$ and its error $F_{b,e,err}$ for a given group of traces. For the particular case $F_{b,e} = 1.8 \pm 0.7$ nN was obtained.

Supplementary Note 4. Geometrical analysis of the simulated junctions

Our simulations of the junction elongation as carried out with perfectly pyramidal gold tips demonstrate significant differences between the geometrical developments of the molecular junctions. Geometrically, the molecules can be characterised by two parameters (Supplementary Table 4). We define the anchor-to-anchor molecular length L as the distance between the centres of the binding atoms of the anchoring groups (i.e. S-S for BT2 and SH2, N-N for PY2 and $NH_2$2) and L_{ar} as the distance between the two farthest atoms of the two aromatic rings. In particular for PY2 $L_{ar} = L$. The values of L_{ar} are very similar for all compounds, illustrating the invariable structure of the aromatic tolane core. Only for BT2 L_{ar} is slightly lower than for other tolanes, probably due to the structural confinement by the dihydrothienyl rings. The binding to the gold can be characterised by a length of a bond between an anchoring atom and Au, $d_{Anchor-Au}$. Supplementary Table 4 lists $d_{Anchor-Au}$ determined from the relaxed geometry of a single-molecule junction where the molecule is bound to the apex atom of only one electrode, i.e. such as S4 in Fig. 3.

We can distinguish 3 general stages of the elongation process. At low distances $z_{Au-Au} < L_{ar}$ (stage I), the orbitals of the aromatic rings of the molecule may overlap with the vacant orbitals of gold (π-Au coupling) and provide an extra electronic coupling to the electrode, thus increasing the junction conductance. We further subdivide stage I into stages corresponding to the π-gold binding geometries with both (Ia) or only one (Ib) aromatic ring(s) stacking onto the sides of the gold tips. At intermediate distances $L_{ar} < z_{Au-Au} < L + 2d_{Anchor-Au}$ (stage II), molecules bridge between the apex gold atoms (anchoring atom-Au coupling). At high distances $z_{Au-Au} > L + 2d_{Anchor-Au}$ (stage III) the single-molecule junction is broken. However, there is a possibility to form a π-π-stacked dimer (π-π coupling) in the presence of two molecules.

The simulated conductance and force traces with 1 and 2 molecules (Fig. 3 and Supplementary Figs 7-9) show clear conductance drops around $z_{Au-Au} = L + 2d_{Anchor-Au}$, i.e. at electrode separations larger than the molecular length plus the length of two anchor-Au bonds. However, only for PY2 the conductance trace demonstrates a transition around L_{ar}. This suggests that the experimental high and middle conductance ranges of PY2 correspond to the geometrical stages I and II, respectively. For the other tolanes, the lack of a clear conductance transition around $z_{Au-Au} = L_{ar}$ might be due to the bigger distance between the aromatic rings and the gold surface, and respectively a weaker electronic coupling, in the stage I. To verify this hypothesis, we analysed for all simulated single-molecule junctions of tolanes the distances from both anchoring groups (N or S) to the nearest gold atoms $d_{Anchor-Au}$ and from both aromatic rings to gold surface $d_{Ring-Au}$. The latter was calculated as the average distance from the 6 atoms of the aromatic ring (C or N) to the nearest gold atom.

Supplementary Figure 10 shows $d_{Anchor-Au}$ and $d_{Ring-Au}$ for both upper and lower gold pyramids (c.f. Figure 6) as a function of the electrode separation z_{Au-Au}. The transition between stages Ia and Ib can be typically seen as a stepwise increase of $d_{Ring-Au}$ for the upper pyramid and a slight decrease for the lower pyramid. They demonstrate that one ring moves away from the upper electrode, and this allows the second ring to assume a better configuration for the binding to the lower electrode. The transition from stage Ib to stage II is represented by an increase of the $d_{Ring-Au}$ for the lower pyramid, which is caused by the detachment of the second ring. The stage II, after short accommodation of the molecule between two apex atoms, is typically characterised by a slight increase of the anchor-gold distance due to the stretching of the bonds. The transition from stage II to stage III, here corresponding to the breaking of the junction, appears as drops of the distances to the lower pyramid and linear increases of the distances to upper pyramid.

The difference between geometries of tolane junctions is highlighted in Supplementary Table 5 that lists average values of $d_{Anchor-Au}$ and $d_{Ring-Au}$ for the aromatic rings bound to the lower electrode (solid and dotted blue curves in Figure 10) during the stage Ib. The difference between PY2 and the other tolanes is clearly indicated by much smaller $d_{Ring-Au}$ and a somewhat higher $d_{Anchor-Au}$ for the former. $d_{Anchor-Au}$ determined for PY2 during the stage Ib is also significantly higher than the value for a relaxed bond (c.f. Supplementary Table 4). These results indicate that during the stage I the nitrogen atom acts as a part of the aromatic ring and as an anchor preferentially binding to the gold apex atoms during the stage II.

Supplementary References

[1] Moreno-García, P. *et al.* Single-molecule conductance of functionalized oligoynes: Length dependence and junction evolution. *J. Am. Chem. Soc.* **135**, 12228–12240 (2013).

[2] Hong, W. *et al.* Single molecular conductance of tolanes: Experimental and theoretical study on the junction evolution dependent on the anchoring group. *J. Am. Chem. Soc.* **134**, 2292–2304 (2012).

[3] Wang, C. *et al.* Oligoyne single molecule wires. *J. Am. Chem. Soc.* **131**, 15647–15654 (2009).

[4] Velizhanin, K. A., Zeidan, T. A., Alabugin, I. V. & Smirnov, S. Single molecule conductance of bipyridyl ethynes: The role of surface binding modes. *J. Phys. Chem. B* **114**, 14189–14193 (2010).

[5] Hybertsen, M. S. *et al.* Amine-linked single-molecule circuits: systematic trends across molecular families. *J. Phys.: Condens. Matter* **20**, 374115 (2008).

[6] Lu, Q. *et al.* From tunneling to hopping: A comprehensive investigation of charge transport mechanism in molecular junctions based on oligo(p-phenylene ethynylene)s. *ACS Nano* **3**, 3861–3868 (2009).

[7] Xing, Y. *et al.* Optimizing single-molecule conductivity of conjugated organic oligomers with carbodithioate linkers. *J. Am. Chem. Soc.* **132**, 7946–7956 (2010).

[8] Wu, S. *et al.* Molecular junctions based on aromatic coupling. *Nat. Nanotechnol.* **3**, 569–574 (2008).

[9] Pobelov, I. V. *et al.* An approach to measure electromechanical properties of atomic and molecular junctions. *J. Phys.: Condens. Matter* **24**, 164210 (2012).

[10] Li, C. *et al.* Charge transport in single Au|alkanedithiol|Au junctions: Coordination geometries and conformational degrees of freedom. *J. Am. Chem. Soc.* **130**, 318–326 (2008).
